# Exploring the psychometric properties of the externalizing spectrum inventory-brief form in a Swedish forensic psychiatric inpatient sample

**DOI:** 10.1186/s12888-023-04609-y

**Published:** 2023-03-21

**Authors:** Johan Berlin, Märta Wallinius, Thomas Nilsson, Malin Hildebrand Karlén, Carl Delfin

**Affiliations:** 1 Lund Clinical Research on Externalizing and Developmental Psychopathology (LU-CRED), Department of Clinical Sciences Lund, Child and Adolescent Psychiatry, Lund University, Lund, Sweden; 2 Centre of Ethics, Law and Mental Health (CELAM), Department of Psychiatry and Neurochemistry, Institute of Neuroscience and Physiology, the Sahlgrenska Academy at University of Gothenburg, Gothenburg, Sweden; 3 Research Department, Regional Forensic Psychiatric Clinic, Växjö, Sweden; 4 Department for Forensic Psychiatry, The National Board of Forensic Medicine, Gothenburg, Sweden; 5 Sahlgrenska University Hospital, University of Gothenburg, Gothenburg, Sweden; 6 Department of Psychology, University of Gothenburg, Gothenburg, Sweden; 7 Institute for Globally Distributed Open Research and Education (IGDORE), Gothenburg, Sweden; 8 Rättspsykiatriska regionkliniken, Box 1223, 351 12 Växjö, Sweden

**Keywords:** ESI-BF, Externalizing spectrum disorders, Forensic psychiatry, Crime, Reliability, Criterion validity

## Abstract

**Background:**

The Externalizing Spectrum Inventory-Brief Form (ESI-BF) [1] is a 160-item self-report instrument designed for the assessment of externalizing psychopathology, yet few studies to date have evaluated its psychometric properties, structural fit, and criterion validity in forensic psychiatric settings.

**Methods:**

Here, we investigated these aspects in a sample of forensic psychiatric inpatients (n = 77) from a maximum-security forensic psychiatric hospital in Sweden. We firstly investigated the reliability. Secondly, using confirmatory factor analysis, the structure of the ESI-BF. And thirdly, using a Bayesian approach, assessed how the three ESI-BF subfactors relate to criterion measures of antisocial behaviors, substance use, and lifetime externalizing spectrum diagnoses.

**Results:**

The ESI-BF demonstrated good to adequate reliability and internal consistency, with all but four facet scales exhibiting α and ω values ≥ 0.80. Average inter-item correlations for the facet scales ranged from 0.31 to 0.74. However, all structural models exhibited poor to mediocre fit, with model fit values for the CFI being 0.66, 0.79 and 0.87 and RMSEA values of 0.14, 0.12 and 0.09. for the unidimensional correlated factors and bifactor model, respectively. Regarding criterion validity, all subscales of the item-based ESI-BF three-factor model exhibited robust correlations with the Life History of Aggression total, aggression and antisocial/consequences subscales, with correlations ranging from 0.29 to 0.55. All ESI-BF subfactors demonstrated robust associations, yet with different externalizing outcomes, lending tentative support to its criterion validity.

**Conclusion:**

Despite remaining ambiguities regarding its structural fit, the ESI-BF may be promising for assessing externalizing psychopathology in forensic psychiatric populations. However, further investigation of the ESI-BF is needed before any firm conclusions can be drawn about its appropriateness in forensic psychiatric settings.

**Supplementary Information:**

The online version contains supplementary material available at 10.1186/s12888-023-04609-y.

## Introduction

The study of externalizing psychopathology dates back to the pioneering work of Thomas Achenbach [2]. Achenbach, who conducted factor analytic studies in child and adolescent psychiatric patients, found support for two major separate factors of symptoms: an internalizing factor, characterized by symptoms of depressive and anxious character and an externalizing factor, characterized by maladaptive and disruptive behaviors and symptoms directed from the individual towards the surrounding environment (e.g., physical and verbal aggression, disobedience, rule breaking and deceitfulness). This classification has, primarily during the last two decades, increasingly been extended into the realm of adult psychopathology [3, 4]. It has gained empirical support in the form of studies documenting the systematic comorbidity of psychiatric diagnoses capturing problems along the externalizing spectrum (e.g., deviant and rule-breaking behaviors, inattention, hyperactivity, impulsivity, alcohol and illicit substance abuse, see e.g., [5–7]). Indeed, categorical psychiatric diagnoses incorporating these symptoms and behaviors, such as conduct disorder (CD), attention deficit hyperactivity disorder (ADHD), antisocial personality disorder (ASPD), and substance use disorders (SUDs), have been found to co-occur within individuals well above what would have been expected had they been unrelated [8–10]. These different manifestations of the externalizing spectrum are suggested to stem from a common, latent vulnerability that links the co-occurrence of externalizing behaviors to a core of impulse control problems, sometimes described as disinhibition or trait impulsivity [11–13]. The way this latent, highly heritable [14, 15], vulnerability is manifested is shaped and influenced by developmental and psychosocial processes, such as experiences of childhood maltreatment and peer group socialization processes in adolescence [11, 16–18].

The systematic co-occurrence of externalizing (as well as internalizing) problems and behaviors, along with perceived limitations of the DSM and ICD approach to classification [19], has led to the development of novel frameworks of classifying psychopathology. These criticism and perceived limitations of the DSM and ICD system have been extensively detailed elsewhere [20–23] and includes the use of categorical diagnoses in a world of dimensional psychopathology, concerns about the reification of diagnostic entities, and the purported failure of these systems to get at the roots of etiological processes of mental disorders, sacrificing validity for reliability. A recent example of an alternative classification framework is the Hierarchical Taxonomy of Psychopathology (HiTOP) [19]. The HiTOP is a large-scale research project seeking to provide a dimensional alternative to the conventional categorical, top-down psychiatric nosology, established on the basis of expert clinician consensus, and as exemplified by the DSM-5 and ICD-10 [24, 25]. In the HiTOP model, the externalizing spectrum is situated second from the top in a hierarchical model, alongside the internalizing spectrum, but below an overarching level of General Psychopathology (see Fig. 1 in Conway et al., [25]).

In forensic psychiatric settings, externalizing problems represent an important treatment target above and beyond the effects of severe mental illnesses. While there are several reliable and well validated measures of major mental disorders such as psychosis, depression and bipolar disorder, the same cannot yet be said for instruments aimed at the comprehensive and dimensional assessment of externalizing problems, although several instruments for specific, narrower, aspects of the externalizing spectrum already exist. From a clinical perspective, externalizing problems and criminogenic needs (e.g., antisocial personality pattern, antisocial associates, substance abuse) may persist well after any severe mental illness has subsided or been successfully treated [26]. Recent research suggests that externalizing problems and criminogenic needs are in fact the primary risk factors for recidivism among persons with severe mental illness, factors that they share with offenders without severe mental illnesses [27–29]. Being able to accurately assess and treat externalizing problems and criminogenic needs, therefore, is crucial, and could aid in further reducing forensic patients’ length of stay and risk for recidivism.

Despite decades of research devoted to delineating the externalizing spectrum, and despite the emergence of several novel frameworks, there are yet few available instruments that capture the associated traits and behaviors in adult populations in a coherent, unified manner. In fact, the Externalizing Spectrum Inventory (ESI; [30]), is, to the best of our knowledge, one of the first instrument developed with the sole purpose of assessing different manifestations of externalizing behaviors, although assessment of externalizing psychopathology has been included as a part of broader instruments (e.g., Achenbach, & Rescorla, 2003 [31]). The ESI offers a comprehensive, dimensional self-report assessment, containing 415 items parsed into 23 fine-grained facets. Its length, however, has proved challenging in practice, resulting in the use of shorter, semi-official versions [32, 33]. To address this, an official, 160-item version was developed, called the Externalizing Spectrum Inventory-Brief Form (ESI-BF) [1]. The ESI-BF aims to provide a more efficient assessment of the externalizing spectrum while retaining the same structure as the original full ESI, with very high correlations at the facet scale level to the full form ESI (*r*_s_ 0.89 –0.98) and high internal consistencies (α > 0.85) across all but one facet scale [1]. An additional goal in the development of the ESI-BF was the creation of three short, item-based subscales indexing different manifestations of the externalizing spectrum. Patrick et al. [1] chose to create these item-based subscales in order to aid research on the externalizing spectrum by allowing researchers to eschew the use of the, sometimes prohibitively extensive, full-length ESI or ESI-BF. The General Disinhibition subfactor (ESI-BF_DIS_) taps the core of the externalizing spectrum and contains 20 items reflecting problematic impulsivity, irresponsibility, lack of planful control, and boredom proneness. The Callous-Aggression subfactor (ESI-BF_AGG_) contains 19 items reflecting dishonesty, deficient empathy, destructiveness, and relational aggression. Finally, the Substance Abuse subfactor (ESI-BF_SUB_) contains 18 items related to recreational and problematic use of alcohol, marijuana, and other substances. Although a bifactor structure of the ESI-BF has emerged as the best fitting model in previous studies [1, 34, 35], the item-based three-factor model (i.e., consisting of the ESI-BF_DIS_, ESI-BF_AGG_ and ESI-BF_SUB_ subfactors), nonetheless appears to be the most widely used variant of the ESI-BF in practice (see e.g., [35–40]). Furthermore, the ESI-BF_DIS_ and ESI-BF_AGG_ scales are also included in the Triarchic Model of Psychopathy [41], which has previously been used in incarcerated samples [42, 43]. Thus, in this article, we focus our assessment of the criterion validity on the item-based ESI-BF three-factor model. The ESI-BF, if found to be valid and reliable in forensic psychiatric settings, could hopefully serve as a measure that will allow us to better assess and, in the end, may help us direct treatment efforts aimed at externalizing problems and thus reduce future risk and recidivism.

Since its introduction, the ESI, the ESI-BF and a previously developed short form, the ESI-100 [44], has been translated into multiple languages and employed in a variety of populations and contexts, including prisoners [45], forensic psychiatric and drug rehabilitation patients [46], and the general population [44]. The ESI is also recommended for assessment of the externalizing spectrum within the HiTOP framework [47]. Nevertheless, so far validation studies of the ESI and the ESI-BF have primarily been conducted in undergraduate [1] and prison samples [33] and studies examining the ESI-BF outside of the North American context are still rare (for exceptions, see [34, 46, 48]). To address these knowledge gaps, and in order to provide a first study exploring the validity of the Swedish translation of the ESI-BF, the current study: (1) presents a descriptive overview and assess the reliability of the ESI-BF, (2) examines the structural fit of three previously proposed models of the ESI-BF [1, 26], and (3) examines how scores on the ESI-BF subfactors relate to early-onset externalizing behaviors (e.g., truancy and bullying), to lifetime aggregate diagnoses of externalizing disorders, as well as to aggressive and antisocial behaviors.

## Methods

### Participants and procedures

Participants were consecutively recruited between the years 2016 and 2020. Eligible patients received oral and written information about the study and gave informed, written consent prior to participation. To be eligible for the study, patients had to be sentenced to forensic psychiatric care (FPC) under the Swedish Forensic Mental Care Act and have an expected stay at the clinic of at least eight weeks. Exclusion criteria included not being sufficiently proficient in speaking and reading Swedish to be able to complete self-report questionnaires and interviews, and/or being deemed as unable to make an informed decision on consent as assessed by the patient’s treating psychiatrist. In practice, this meant that patients who demonstrated severe neuropsychiatric or intellectual disabilities, or acute and severe psychotic symptoms were excluded. After participation, participants were given a gift card for shopping at a clinic kiosk or local mall (~$10). In total, 277 patients were available at the clinic at the time of inclusion, of which 93 were excluded on basis of the exclusion criteria. Among the eligible patients, 101 provided informed consent, of which three withdrew their consent prior to participation (overall participation rate: 56%). Out of these 98 participants, 77 patients had the requisite complete data to be included in the analyses for the current study.

The participants in the current study were thus 77 forensic psychiatric inpatients from a Swedish, maximum-security forensic psychiatric hospital. The majority of participants were male (n = 66, 86%), the mean age was 36 years (SD = 10.5, range = 20–62), and about one in five (n = 16, 21%) had not graduated from primary school. As per Swedish legislation, all participants had been sentenced to FPC after committing a crime [49]. The most common psychiatric diagnoses were schizophrenia spectrum disorders or other psychotic disorders (n = 38, 49% as primary diagnosis and n = 6, 8% as secondary diagnosis), autism spectrum disorders (n = 11, 14% as primary diagnosis and n = 7, 9% as secondary diagnosis), antisocial personality disorder (n = 7, 9% as primary diagnosis and n = 9, 12% as secondary diagnosis), and substance use disorders (n = 2, 3% as primary diagnosis and n = 25, 32% as secondary diagnosis). Comorbidities within the externalizing spectrum, such as criminality, were common, and the sample characteristics corresponded well with Swedish forensic psychiatric patients in general [50, 51]. Additional details about the psychosocial background and criminological and clinical characteristics of the full sample (N = 98) can be in found in Laporte et al. [49].

### Measures

#### The Externalizing Spectrum Inventory-Brief Form

The current study used the Swedish (official and back-translated) version of the ESI-BF. In the ESI-BF, 160 items are rated using self-report on a scale from 0 (Not true at all) to 3 (Completely true), with possible scores ranging from 0 to 60 for the ESI-BF_DIS_, 0 to 57 ESI-BF_AGG_, and 0 to 54 for the ESI-BF_SUB_ subfactors. A list of the 23 facet scales can be found in Table 1 and further details on the items included in each facet scale is found in the supplemental material to Patrick et al., 2013 [1]. In the current study, the paper and pencil version of the ESI-BF was administered in the presence of a data collector, allowing participants to ask questions or gain assistance in interpreting the items if necessary.

#### The Life History of Aggression

The Life History of Aggression (LHA; [52, 53]) was used as an assessment of lifetime aggressive and antisocial behaviors. The 11 LHA items, which can be completed through self-report or by an assessor, are summed in a total score (LHA_TOT_), with possible scores ranging from 0 to 55, and divided into three separate subscales: Aggression (LHA_AGG_), Consequences/Antisocial Behavior (LHA_ANTI_), and Self-directed Aggression (LHA_SELF_). Each item is scored, based on the aggregated lifetime occurrence of the behaviors in question, on scale from 0 to 5. A score of 0 denotes that the behavior has never occurred while a score of 5 indicates that the behavior has occurred so frequently as to be innumerous. The LHA_AGG_ subscale consists of 5 items related to temper tantrums, physical fights, verbal aggression, physical assaults on people or animals, and assaults on property, with possible subscale scores ranging from 0 to 25. The LHA_ANTI_ subscale consists of 4 items referring to school disciplinary problems, problems with supervisors at work, and antisocial behaviors with and without police involvement, with possible subscale scores ranging from 0 to 20. The LHA_SELF_ subscale quantifies self-injurious behavior and suicide attempts in two items, with possible subscale scores ranging from 0 to 10. The LHA, as rated by the data collectors, was based on the total information available from patients’ self-reports, interviews and file reviews. All LHA assessments were reviewed by the data collectors and a senior researcher (author MW) to reach a consensus score with the senior researcher having the final say in the interpretation of the sources. When information from patient self-reports and file reviews conflicted, a higher score was awarded if indicated in the file material.

#### Clinical and criminological measures

File data on lifetime history of psychiatric disorders and criminological variables were collected using a structured data collection form, extracting information from the patients’ medical records (incl. current FPC and previous psychiatric care episodes) and forensic psychiatric investigations. These data were, together with participants’ self-reports on criminological background from interviews, used to create fourteen binary variables, depicting the participants’ lifetime history of early-onset disruptive behaviors lifetime externalizing disorders and aggressive and antisocial behaviors. An overview of these variables, along with a brief description on how they were created, is available in the Supplemental Material (see supplementary material 1).

Using file and self-report data, we obtained information on participants’ age at onset of criminality and age at first sentence (the latter has a lower bound of fifteen years, the age of criminal responsibility in Sweden), as well as their total number of sentences (with the total number of prison sentences being a separate variable). Finally, an ordinal variable was created which counted lifetime instances of externalizing diagnoses for each participant. Included diagnoses were: oppositional defiant disorder (ODD), CD, ADHD, intermittent explosive disorder, SUDs, ASPD, kleptomania, as well as both other specified and unspecified disruptive, impulse-control, and conduct disorders.

### Analyses

All data processing and statistical analysis was carried out using the R statistical language, version 4.1.1 [54]. Several packages from the Tidyverse [55] were used for intermediate data processing, and all code is freely and publicly available on the Open Science Framework (https://osf.io/kd9vz/, 10.17605/OSF.IO/KD9VZ).

#### Descriptive overview, reliability analysis and internal consistency

Means, standard deviations (SDs), range, inter-item correlations, and Cronbach’s α and McDonald’s ω reliability coefficients, in line with recent recommendations [56], were computed for all facets and subfactors of the ESI-BF and all LHA subscales. The average inter-item correlation, using Spearman’s $\rho$, was calculated in order to assess the extent to which scores on one item are correlated with scores on all other items in a particular ESI-BF facet or subfactor. In essence, a high inter-item correlation suggests that the item may be redundant and does not contribute anything unique to the construct, while a low inter-item correlation suggests that the item may not be representative of the construct. Typically, values between 0.15 and 0.50 are considered acceptable [57].

#### Confirmatory factor analysis

**Fig. 1:**
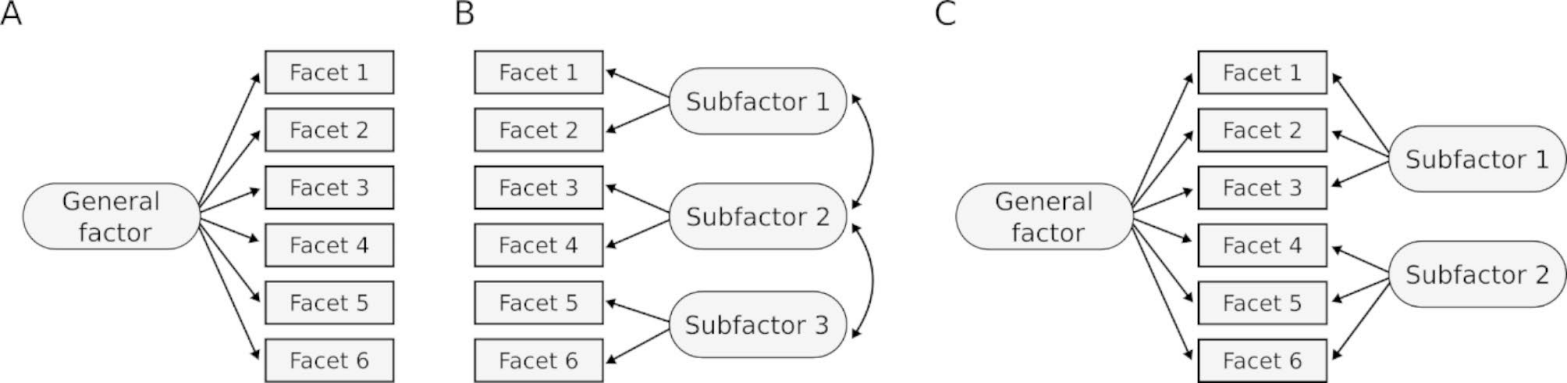
Structural models of the ESI-BF

Confirmatory factor analysis (CFA), with scores on the 23 ESI-BF facets as units of measurement, was used to evaluate the structure of the ESI-BF. The R package lavaan [58] was used to fit three different structural models, following previous research [1, 30, 33, 34]: (1) a unidimensional model, (2) a correlated factors model, and (3) a bifactor (or hierarchical) model. In the unidimensional model a single, general factor (λ_G_), called “disinhibition” or “externalizing” by Patrick et al. [1], is theorized to explain the variance across all facets. In the correlated factors model, facets are grouped by theorized similarities and function as indicators for three different latent factors (λ_DIS_, λ_AGG_, λ_SUB_) that mimic the ESI-BF subfactors. In this model, the latent factors are allowed to be correlated with each other. Finally, in the bifactor model, all facets load on a single, general factor (λ_G_), while residual variance of certain facets not accounted for by the general factor load on two separate factors (λ_RAGG_ and λ_RSUB_). Note that two facets, Blame Externalization and Rebelliousness, were omitted from the correlated factors model following [1], and that in the bifactor model, the two subfactors were orthogonal (i.e., uncorrelated) to the general factor and to each other. See Fig. 1 for a conceptual visualization of each model.

*Three structural models of the ESI-BF: (A) a unidimensional model, (B) a correlated factors model, and (C) a bifactor (or hierarchical) model.*

Given the small sample size, and since non-normal data and model misfit are, in practice, almost always present to some degree [59], all CFAs where modelled using a maximum likelihood estimator with Huber-White robust standard errors. Model fit was evaluated using the Akaike information criterion (AIC), the sample-size adjusted Bayesian information criterion (SABIC), the comparative fit index (CFI), the Tucker-Lewis index (TLI), the root mean square error of approximation (RMSEA), and the standardized root mean square residual (SRMR). When possible, robust variants of these fit measures were used. The AIC and SABIC may only be compared for models using the same data and variables, and both penalize for adding additional parameters to the model, but the SABIC adjusts the penalty based on sample size. Thus, the AIC and SABIC values of the correlated factors model are not directly comparable to the other two models. In both cases, lower values indicate better model fit in the sense that the model with the lowest value is more likely to accurately predict new data. Cut-offs recommended by Little [60] were used for remaining measures: For CFI and TLI, values below 0.85 indicate poor fit, values between 0.85 and 0.90 indicate mediocre fit, values between 0.90 and 0.95 indicate acceptable fit, and between 95 and 0.99 indicate very good fit. For RMSEA and SRMR, values > 0.10 indicate poor fit, values between 0.10 and 0.08 indicate mediocre fit, values between 0.08 and 0.05 indicate acceptable fit, values between 0.05 and 0.02 indicate good fit, and values below 0.01 indicate great fit.

#### Criterion validity

We opted for a fully Bayesian approach, and the statistical models were specified using the R package “brms” [61]. All Bayesian priors were chosen to be robust and weakly informative, thus having negligible impact on obtained estimates while still providing moderate regularization by gently pushing all estimates towards zero [62]. For dichotomous variables, we examined group differences in scores on the three ESI-BF subfactors using a robust linear regression approach. Robustness was achieved by using a Student’s T likelihood [63], which ameliorates the possible influence of outliers. We also allowed for unequal variances between groups. For continuous variables, we examined zero-order correlations with ESI-BF subfactor scores (correlations with bifactor model scores are available in the Supplemental Material (see supplementary material 5), again using a robust linear regression approach, with a multivariate Student’s T likelihood and a correlation matrix drawn from an LKJ (2) prior [64]. Model sampling was carried out using 12 chains with 4,000 iterations each, after 1,000 warm-up iterations were discarded.

Results from group comparisons are presented as posterior medians of the estimated difference as well as of the bias-corrected standardized difference (denoted $\stackrel{\prime }{\delta }$, also known as Cohen’s d with Hedges’s g correction [65]). Results from correlation models are presented as the posterior median of the estimated correlation ($\rho$). We suggest that readers interpret relevant correlation estimates in light of recent research on the typical effect sizes in psychological research (e.g., [66, 67]). Thus, r = 0.1 may indicate a small but, in the long run, potentially consequential effect, r = 0.2 may indicate a medium effect that might be of some explanatory and practical use, and r = 0.3 might indicate an effect that is potentially large both in the short and long run. Larger effects, r > 0.4, are likely exaggerated and not replicable in larger sample sizes. Note, however, that these guidelines may not be relevant when large correlations are expected, such as inter-correlations between two similar instruments. In addition, all median estimates are reported along with 90% highest density intervals (HDIs), presented within square brackets. Our choice of a 90% interval is in line with recommendations for how to quantify uncertainties in everyday language [68], and thus a 90% HDI may be interpreted such that there is a 90% probability, or “very likely” in non-mathematical terms, that the parameter estimate falls within its range [69]. To aid in interpretation, results where the 90% HDI does not contain zero are referred to as “robust”.

## Results

### Reliability analysis

Average inter-item correlations ranged between 0.31 and 0.74, with the highest values observed for the Marijuana Use, Marijuana Problems, Drug Problems, and Boredom Proneness facets. Overall, α and ω estimates were very similar and ranged from 0.61 to 0.96. Notably, α and ω values for the Alienation facet were 0.61 and 0.67, respectively, while the majority of remaining facets were ≥ 0.80. Details, along with descriptive measures, are presented in Table 1.

**Table 1:** Descriptive overview and reliability measures of the ESI-BF (N = 77)

		N_items_	*M*	*Md*	SD	Range	*α*	*ω*	*M*_r_
**Subfactors**									
	General Disinhibition	20	25.56	26	14.04	2–60	0.90	0.90	0.31
	Callous Aggression	19	15.40	13	12.11	0–51	0.87	0.91	0.33
	Substance Abuse	18	26.25	29	14.48	3–50	0.91	0.91	0.35
**Facet**									
	Alcohol Problems	9	8.00	5	8.32	0–27	0.92	0.92	0.55
	Alcohol Use	9	15.00	15	6.45	0–27	0.81	0.81	0.31
	Alienation	3	4.45	4	2.56	0–9	0.61	0.67	0.35
	Blame Externalization	4	6.34	7	3.86	0–12	0.85	0.85	0.59
	Boredom Proneness	4	6.96	8	4.17	0–12	0.88	0.89	0.65
	(Lack of) Dependability	7	4.82	4	4.29	0–19	0.81	0.83	0.39
	Destructive Aggression	7	5.03	4	5.25	0–20	0.80	0.80	0.35
	Drug Problems	11	15.14	15	12.98	0–33	0.96	0.96	0.68
	Drug Use	6	9.84	12	6.38	0–18	0.88	0.89	0.56
	(Lack of) Empathy	11	9.62	8	8.16	0–33	0.89	0.89	0.42
	Excitement Seeking	6	6.25	5	5.35	0–18	0.86	0.86	0.51
	Fraud	6	3.53	3	4.06	0–17	0.75	0.76	0.35
	(Lack of) Honesty	5	2.84	2	2.86	0–13	0.73	0.75	0.37
	Impatient Urgency	5	8.30	9	4.76	0–15	0.88	0.88	0.60
	Irresponsibility	10	11.05	11	7.73	0–27	0.82	0.82	0.30
	Marijuana Problems	7	5.84	1	7.22	0–21	0.93	0.94	0.68
	Marijuana Use	7	11.18	13	8.60	0–21	0.95	0.95	0.74
	Physical Aggression	8	11.95	12	6.95	0–24	0.85	0.85	0.41
	(Lack of) Planful control	6	4.96	4	4.22	0–17	0.85	0.85	0.49
	Problematic Impulsivity	7	11.77	13	6.31	0–21	0.88	0.88	0.51
	Rebelliousness	6	7.27	7	5.45	0–18	0.86	0.87	0.51
	Relational Aggression	8	6.16	4	5.95	0–27	0.70	0.83	0.36
	Theft	8	11.12	11	7.67	0–24	0.88	0.88	0.48

*Note*. *M*_r_, average inter-item correlation

α, Cronbach’s Alpha

ω, McDonald’s Omega

### Confirmatory factor analysis

Relative fit indexes indicated poor fit for the unidimensional (CFI = 0.66, TLI = 0.63) and correlated factors (CFI = 0.79, TLI = 0.76) models, and mediocre fit for the bifactor model (CFI = 0.87, TLI = 0.84). Similarly, absolute fit indexes indicated poor fit for the unidimensional (RMSEA = 0.14, 95% CI [0.13, 0.16], SRMR = 0.11) and correlated factors (RMSEA = 0.12, 95% CI [0.10, 0.14]), SRMR = 0.11) models, and mediocre to acceptable fit for the bifactor model (RMSEA = 0.09, 95% CI [0.08, 0.11], SRMR = 0.07). Finally, both comparative fit indexes, as well as AIC and SABIC, favored the bifactor model. Details are presented in Table 3.

**Table 2:** Fit statistics from confirmatory factor analyses (N = 77)

Model	k	AIC	SABIC	CFI_r_	TLI_r_	RMSEA [95% CI]	SRMR
Unidimensional	46	10,504.10	10,466.90	0.66	0.63	0.14 [0.13, 0.16]	0.11
Correlated factors	45	9,554.97	9,518.58	0.79	0.76	0.12 [0.1, 0.14]	0.11
Bifactor	69	10,294.67	10,238.88	0.87	0.84	0.09 [0.08, 0.11]	0.07

*Note*. k, number of free parameters; AIC, Akaike information criterion; SABIC, sample size adjusted Bayesian information criterion; CFI_r_, Comparable fit index, robust version; TLI_r_, Tucker-Lewis index, robust version; RMSEA, root mean square error of approximation; SRMR, standardized root mean square residual

The latent factors in the correlated factors model exhibited relatively strong inter-correlations, with *r* = 0.87 between λ_DIS_ and λ_AGG_, *r* = 0.65 between λ_DIS_ and λ_SUB_, and 0.53 between λ_AGG_ and λ_SUB_. Standardized factor loadings, standard errors, and residual variances for each model are presented in Table 2.

**Table 3:** Parameter estimates from confirmatory factor models (N = 77)

	Unidimensional model		Correlated factors model				Bifactor model			
Facet	λ_G_	Θ	λ_DIS_	λ_AGG_	λ_SUB_	Θ	λ_G_	λ_RAGG_	λ_RSUB_	Θ
Alcohol Problems	0.52 (0.10)	0.73 (0.10)	0	0	0.44 (0.11)	0.81 (0.09)	0.54 (0.09)	0	0.05 (0.11)	0.70 (0.10)
Alcohol Use	0.45 (0.10)	0.80 (0.09)	0	0	0.42 (0.09)	0.83 (0.07)	0.41 (0.11)	0	0.14 (0.13)	0.81 (0.09)
Alienation	0.49 (0.09)	0.76 (0.09)	0.5 (0.10)	0	0	0.75 (0.10)	0.60 (0.09)	-0.34 (0.13)	0	0.52 (0.14)
Blame Externalization	0.47 (0.09)	0.78 (0.09)	-	-	-	-	0.56 (0.08)	-0.36 (0.17)	0	0.56 (0.16)
Boredom Proneness	0.57 (0.09)	0.67 (0.10)	0.56 (0.09)	0	0	0.69 (0.10)	0.56 (0.09)	0.09 (0.14)	0	0.68 (0.10)
(Lack of) Dependability	0.60 (0.10)	0.64 (0.12)	0.65 (0.09)	0	0	0.57 (0.12)	0.59 (0.11)	0.27 (0.20)	0	0.58 (0.12)
Destructive Aggression	0.64 (0.07)	0.59 (0.09)	0	0.71 (0.08)	0	0.50 (0.11)	0.63 (0.07)	0.28 (0.17)	0	0.52 (0.10)
Drug Problems	0.80 (0.05)	0.36 (0.08)	0	0	0.89 (0.03)	0.20 (0.05)	0.72 (0.06)	0	0.51 (0.08)	0.21 (0.04)
Drug Use	0.72 (0.07)	0.48 (0.09)	0	0	0.91 (0.03)	0.16 (0.06)	0.62 (0.07)	0	0.66 (0.07)	0.19 (0.06)
(Lack of) Empathy	0.39 (0.11)	0.85 (0.08)	0	0.56 (0.13)	0	0.68 (0.14)	0.30 (0.11)	0.63 (0.19)	0	0.51 (0.23)
Excitement Seeking	0.73 (0.07)	0.46 (0.11)	0	0.75 (0.08)	0	0.43 (0.12)	0.69 (0.08)	0.34 (0.11)	0	0.41 (0.10)
Fraud	0.72 (0.05)	0.48 (0.08)	0.74 (0.06)	0	0	0.45 (0.09)	0.69 (0.07)	0.30 (0.15)	0	0.44 (0.09)
(Lack of) Honesty	0.43 (0.12)	0.81 (0.10)	0	0.53 (0.10)	0	0.72 (0.11)	0.37 (0.13)	0.50 (0.20)	0	0.61 (0.19)
Impatient Urgency	0.56 (0.10)	0.69 (0.11)	0.61 (0.09)	0	0	0.63 (0.11)	0.59 (0.09)	0.23 (0.11)	0	0.60 (0.10)
Irresponsibility	0.78 (0.05)	0.38 (0.08)	0.80 (0.05)	0	0	0.36 (0.08)	0.80 (0.05)	0	0.11 (0.09)	0.35 (0.08)
Marijuana Problems	0.53 (0.09)	0.72 (0.10)	0	0	0.75 (0.05)	0.44 (0.08)	0.43 (0.09)	0	0.66 (0.06)	0.38 (0.06)
Marijuana Use	0.61 (0.10)	0.63 (0.12)	0	0	0.89 (0.03)	0.20 (0.06)	0.47 (0.09)	0	0.84 (0.06)	0.06 (0.05)
Physical Aggression	0.64 (0.07)	0.59 (0.09)	0	0.7 (0.08)	0	0.52 (0.11)	0.59 (0.08)	0.35 (0.18)	0	0.53 (0.13)
(Lack of) Planful Control	0.58 (0.10)	0.66 (0.12)	0.66 (0.09)	0	0	0.57 (0.12)	0.64 (0.08)	0.09 (0.20)	0	0.59 (0.11)
Problematic Impulsivity	0.70 (0.07)	0.50 (0.09)	0.77 (0.06)	0	0	0.40 (0.09)	0.80 (0.06)	0	-0.20 (0.10)	0.32 (0.10)
Rebelliousness	0.86 (0.03)	0.27 (0.05)	-	-	-	-	0.83 (0.04)	0.12 (0.12)	0	0.30 (0.06)
Relational Aggression	0.64 (0.06)	0.59 (0.08)	0	0.77 (0.06)	0	0.41 (0.09)	0.63 (0.06)	0.42 (0.17)	0	0.43 (0.11)
Theft	0.77 (0.05)	0.41 (0.08)	0.72 (0.07)	0	0	0.48 (0.10)	0.72 (0.06)	0	0.25 (0.08)	0.42 (0.08)

*Note*. The general factor in the unidimensional and bifactor models is labelled λ_G_, loadings for the correlated factors model are labelled λ_DIS_, λ_AGG_, λ_SUB_, mimicking the item-based subfactors of the ESI-BF, and loadings for the two residual factors in the bifactor model are labelled λ_RAGG_, and λ_RSUB_. Residual variances are labelled Θ, with standard errors shown in parentheses. Values of 0 are fixed and not estimated, and dashes denote facets omitted from the model

### Criterion validity

#### Group differences

Forensic psychiatric patients with repeated truancy, problematic alcohol use/abuse, and repeated substance use, as well as those with multiple sentences for property crimes, multiple sentences for drug-related crimes, and any sentence for financial crimes had robustly higher scores on the ESI-BF_DIS_ scale than those without. Estimated differences in raw ESI-BF_DIS_ score for these measures ranged from 6.32 [0.57, 11.99] for any sentence for financial crimes to 11.51 [6.58, 16.37] for problematic alcohol use/abuse. Full details, with descriptive measures, are presented in Table 4. Additionally, results from analyses of regression-based estimates of factor scores from the facet-based bifactor model are available in the Supplemental Material (see supplementary material 2–4).

**Table 4:** Descriptive statistics (means and standard deviations) and posterior medians of the estimated difference and effect size for the ESI-BF General disinhibition subfactor (N = 77)

Measure	*M*_yes_ (SD)	*M*_no_ (SD)	Est. diff. [90% HDI]	Est. $\stackrel{\prime }{\delta }$ [90% HDI]
Repeated truancy	28.62 (14.69)	19.89 (10.86)	**8.99 [3.95, 13.99]**	0.68 [0.29, 1.08]
Repeated bullying	24.71 (14.17)	25.80 (14.11)	-1.07 [-7.88, 5.73]	-0.08 [-0.55, 0.42]
Any violence against caregiver	28.48 (14.43)	23.36 (13.48)	5.42 [-0.03, 11.00]	0.39 [0.00, 0.8]
Excessive alcohol use	30.29 (14.21)	18.91 (10.88)	**11.51 [6.58, 16.37]**	0.89 [0.50, 1.31]
Excessive substance use	28.05 (14.18)	17.39 (10.12)	**10.86 [5.64, 16.05]**	0.82 [0.40, 1.23]
Any sentence for deadly violence	24.25 (10.17)	26.02 (15.21)	-1.45 [-6.57, 3.84]	-0.10 [-0.48, 0.26]
Multiple sentences for assault	26.15 (13.99)	24.59 (14.31)	1.78 [-3.84, 7.57]	0.13 [-0.27, 0.54]
Multiple sentences for other violence crimes	25.00 (13.94)	27.69 (14.65)	-2.50 [-9.76, 4.42]	-0.18 [-0.68, 0.31]
Any sentence for sexual crimes^1^	20.90 (10.05)	26.20 (14.57)	-5.18 [-11.53, 1.54]	-0.37 [-0.84, 0.10]
Multiple sentences for theft or damage to property	28.19 (14.13)	20.08 (12.40)	**8.70 [3.18, 13.88]**	0.66 [0.23, 1.07]
Any sentence for economics-related crimes	29.82 (12.83)	23.85 (14.24)	**6.32 [0.57, 11.99]**	0.46 [0.03, 0.87]
Any sentence for traffic-related crimes	26.51 (14.90)	23.46 (11.93)	3.46 [-1.97, 8.99]	0.25 [-0.14, 0.65]
Multiple sentences for narcotics-related crimes	28.24 (14.16)	16.78 (9.50)	**11.66 [6.80, 16.8]**	0.89 [0.48, 1.30]
Multiple sentences for weapons-related crimes	28.07 (12.53)	24.04 (14.79)	4.58 [-0.83, 10.05]	0.33 [-0.07, 0.72]

*Note*. ^1^ N = 76 for sexual crimes. ESI-BF, Externalizing Spectrum Inventory-Brief Form; HDI, highest density interval; $\stackrel{\prime }{\delta }$, bias-corrected standardized mean difference. Estimated differences for which the 90% HDI does not contain zero are highlighted in bold

Forensic psychiatric patients with repeated truancy, repeated bullying of others, any violence against their caregiver, as well as those with multiple sentences for assaults, drug-related crimes, and weapon-related crimes had robustly higher scores on the ESI-BF_AGG_ scale than those without. In addition, those with any sentence for a sexual crime had robustly lower scores on the ESI-BF_AGG_ than those without. Estimated differences in raw ESI-BF_AGG_ score for these measures ranged from 4.91 [0.77, 8.87] for repeated truancy to 6.37 [1.96, 10.69] for multiple sentences for weapon-related crimes. Full details, with descriptive measures, are presented in Table 5.

**Table 5:** Descriptive statistics (means and standard deviations) and posterior medians of the estimated difference and effect size for the ESI-BF Callous-aggression subfactor (N = 77)

Measure	*M*_yes_ (SD)	*M*_no_ (SD)	Est. diff. [90% HDI]	Est. $\stackrel{\prime }{\delta }$ [90% HDI]
Repeated truancy	17.30 (13.09)	11.89 (9.29)	**4.91 [0.77, 8.87]**	0.48 [0.08, 0.87]
Repeated bullying	19.47 (11.39)	14.25 (12.15)	**5.45 [1.10, 9.83]**	0.63 [0.10, 1.21]
Any violence against caregiver	18.64 (12.19)	12.98 (11.6)	**6.10 [2.07, 10.12]**	0.69 [0.21, 1.20]
Excessive alcohol use	16.22 (12.08)	14.25 (12.26)	3.02 [-0.87, 6.97]	0.33 [-0.11, 0.75]
Excessive substance use	16.41 (11.99)	12.11 (12.27)	4.49 [-0.43, 9.30]	0.47 [-0.04, 1.02]
Any sentence for deadly violence	16.25 (11.83)	15.11 (12.30)	1.85 [-2.82, 6.51]	0.19 [-0.29, 0.69]
Multiple sentences for assault	17.6 (12.47)	11.76 (10.73)	**5.87 [1.87, 9.62]**	0.64 [0.20, 1.09]
Multiple sentences for other violence crimes	15.57 (12.43)	14.75 (11.20)	0.41 [-4.62, 5.44]	0.04 [-0.46, 0.55]
Any sentence for sexual crimes^1^	9.40 (7.72)	16.15 (12.47)	**-5.44 [-10.82, -0.12]**	-0.53 [-1.09, -0.02]
Multiple sentences for theft or damage to property	16.48 (12.38)	13.16 (11.47)	3.16 [-1.21, 7.43]	0.32 [-0.13, 0.75]
Any sentence for economics-related crimes	17.91 (11.96)	14.40 (12.14)	4.15 [-0.35, 8.56]	0.46 [-0.06, 0.98]
Any sentence for traffic-related crimes	16.13 (11.45)	13.79 (13.59)	3.18 [-1.84, 7.94]	0.34 [-0.19, 0.90]
Multiple sentences for narcotics-related crimes	16.8 (11.96)	10.83 (11.81)	**6.37 [1.96, 10.69]**	0.70 [0.18, 1.22]
Multiple sentences for weapons-related crimes	19.07 (12.43)	13.19 (11.49)	**6.32 [1.88, 10.67]**	0.69 [0.20, 1.19]

*Note*. ^1^ N = 76 for sexual crimes. ESI-BF, Externalizing Spectrum Inventory-Brief Form; HDI, highest density interval; $\stackrel{\prime }{\delta }$, bias-corrected standardized mean difference. Estimated differences for which the 90% HDI does not contain zero are highlighted in bold

Forensic psychiatric patients with repeated truancy, problematic alcohol use/abuse, and repeated substance use, as well as those with multiple sentences for property crimes, drug-related crimes, weapon-related crimes, and with any sentence for financial and traffic crimes had robustly higher scores on the ESI-BF_SUB_ scale than those without. Estimated differences in raw ESI-BF_SUB_ scores for these measures ranged from 7.47 [2.09, 13.21] for any sentence for financial crimes to 23.43 [20.07, 26.83] for multiple sentences for drug-related crimes. Full details, with descriptive measures, are presented in Table 6.

**Table 6:** Descriptive statistics (means and standard deviations) and posterior medians of the estimated difference and effect size for the ESI-BF Substance abuse subfactor (N = 77)

Measure	*M*_yes_ (SD)	*M*_no_ (SD)	Est. diff. [90% HDI]	Est. $\stackrel{\prime }{\delta }$ [90% HDI]
Repeated truancy	30.4 (13.78)	18.56 (12.67)	**12.58 [7.12, 17.88]**	0.94 [0.51, 1.37]
Repeated bullying	26.65 (14.55)	26.13 (14.58)	0.54 [-6.5, 7.69]	0.04 [-0.43, 0.52]
Any violence against caregiver	28.97 (14.38)	24.2 (14.38)	5.21 [-0.53, 10.9]	0.36 [-0.03, 0.76]
Excessive alcohol use	31.69 (13.53)	18.59 (12.28)	**13.69 [8.51, 18.82]**	1.04 [0.63, 1.48]
Excessive substance use	31.44 (12.12)	9.22 (5.95)	**23.33 [19.7, 26.97]**	2.18 [1.67, 2.76]
Any sentence for deadly violence	24.8 (14.62)	26.75 (14.52)	-2 [-8.47, 4.62]	-0.14 [-0.57, 0.32]
Multiple sentences for assault	28.1 (13.8)	23.17 (15.28)	5.25 [-0.83, 11.23]	0.36 [-0.06, 0.77]
Multiple sentences for other violence crimes	25.93 (13.99)	27.44 (16.65)	-1.57 [-9.73, 6.42]	-0.11 [-0.64, 0.43]
Any sentence for sexual crimes^1^	19.6 (14.77)	27.08 (14.33)	-7.96 [-16.91, 1.05]	-0.55 [-1.16, 0.07]
Multiple sentences for theft or damage to property	28.75 (14.09)	21.04 (14.15)	**8.1 [2.06, 13.99]**	0.57 [0.14, 0.99]
Any sentence for economics-related crimes	31.36 (11.81)	24.2 (15.03)	**7.47 [2.09, 13.21]**	0.52 [0.13, 0.92]
Any sentence for traffic-related crimes	29.02 (12.91)	20.12 (16.1)	**9.75 [3.02, 16.14]**	0.69 [0.2, 1.16]
Multiple sentences for narcotics-related crimes	31.54 (12.05)	8.89 (5.05)	**23.43 [20.07, 26.83]**	2.21 [1.71, 2.73]
Multiple sentences for weapons-related crimes	30.76 (12.45)	23.52 (15.05)	**7.72 [1.97, 13.01]**	0.54 [0.15, 0.94]

*Note*. ^1^ N = 76 for sexual crimes. ESI-BF, Externalizing Spectrum Inventory-Brief Form; HDI, highest density interval; $\stackrel{\prime }{\delta }$, bias-corrected standardized mean difference. Estimated differences for which the 90% HDI does not contain zero are highlighted in bold

#### Correlations

All three ESI-BF subfactors showed robust associations with scores on the LHA_TOT_, LHA_AGG_, and LHA_ANTI_ subscales, with estimates ranging from 0.29 to 0.55. Notably, ESI-BF_DIS_ consistently showed the highest estimates, ranging from 0.44 to 0.55, and was the sole ESI-BF subfactor to exhibit a robust (0.21) association with scores on the LHA_SELF_ subscale. All three ESI-BF subfactors were negatively associated with age at first crime and age at first sentence, with estimates ranging from − 0.16 to − 0.27, but the association between ESI-BF_SUB_ and age at first crime was not robustly below zero. All three ESI-BF subfactors were also positively associated with the total number of sentences, ranging from 0.18 to 0.20, although, again, neither of these estimates were robustly above zero. Similar results were observed for the total number of prison sentences, yet estimates here were both smaller and less robust. Full details, along with descriptive measures, are presented in Table 7.

**Table 7:** Descriptive statistics (N, means, standard deviations, medians, and range) and posterior medians of the estimated correlation with ESI-BF subfactors for Life History of Aggression and criminological measures

	Descriptive statistics				Est. corr. [90% HDI]		
Measure	*N*	*M* (SD)	*Md*	Range	ESI-BF_DIS_	ESI-BF_AGG_	ESI-BF_SUB_
LHA_TOT_	75	34.6 (10.51)	37	7–50	**0.55 [0.42, 0.68]**	**0.33 [0.15, 0.50]**	**0.40 [0.23, 0.56]**
LHA_AGG_	77	17.58 (5.46)	18	4–25	**0.44 [0.28, 0.59]**	**0.34 [0.18, 0.52]**	**0.29 [0.11, 0.46]**
LHA_ANTI_	76	13.92 (5.25)	15	0–20	**0.53 [0.39, 0.66]**	**0.34 [0.17, 0.51]**	**0.49 [0.34, 0.63]**
LHA_SELF_	75	3.27 (3.06)	3	0–10	**0.21 [0.01, 0.38]**	− 0.10 [-0.29, 0.10]	0.02 [-0.18, 0.21]
Age at first crime	73	14.96 (7.10)	14	6–47	**− 0.27 [-0.46, − 0.08]**	**− 0.24 [-0.44, − 0.04]**	− 0.16 [-0.37, 0.03]
Age at first sentence	77	22.73 (8.22)	19	15–50	**− 0.24 [-0.42, − 0.06]**	**− 0.23 [-0.42, − 0.05]**	**− 0.27 [-0.45, − 0.09]**
Total number of sentences	77	7.43 (8.23)	4	1–50	0.20 [0.00, 0.39]	0.18 [-0.02, 0.39]	0.19 [0.00, 0.39]
Total number of prison sentences	77	1.96 (5.14)	0	0–38	0.08 [-0.14, 0.28]	0.18 [-0.03, 0.37]	0.12 [-0.08, 0.32]

*Note*. ESI-BF_DIS_, ESI-BF_AGG_, and ESI-BF_SUB_ represent item-based subfactors from the Externalizing Spectrum Inventory-Brief Form; HDI, highest density interval. Estimated correlations for which the 90% HDI does not contain zero are highlighted in bold

## Discussion

This study is one of the first to examine the psychometric properties and criterion validity of the ESI-BF in a forensic psychiatric inpatient sample. Furthermore, it is the first study to our knowledge to attempt to validate the ESI-BF in Swedish context and one of the first few to investigate the ESI-BF outside of the United States. Overall, we found the basic psychometric properties to be satisfactory, while the structural models fared less well. In regard to criterion validity, however, the ESI-BF showed some promise as a measure of externalizing problems in this population.

In relation to our first aim, we found that the basic psychometric properties of the ESI-BF held up well, with α-values for all but four facet scales (Alienation, Fraud, Honesty, Relational Aggression) falling within the “satisfactory” range (α ≥ 0.80) [70], and with similarly consistently high ω-values; only three facet scales (Alienation, Fraud, Honesty) had ω-values below 0.80. Notably, since ω-values are generally considered to rely on more plausible model assumptions for psychological attributes than α-values [71], they may be considered a more precise estimate of an instrument’s basic psychometric properties. Average inter-item correlation values fell within the range of values that indicates that the items sample the intended construct in a way that is neither to broad nor too narrow. Four facet scales, however, (Marijuana Use, Marijuana Problems, Drug Problems, Boredom Proneness) stood out with regards to elevated values, indicating that these facet scales may contain redundant items and tap their respective domain to narrowly. Three of them (Marijuana Use, Marijuana Problems, Boredom Proneness) exhibited similarly elevated values in recent work by Soe-Agnie et al. [34], also carried out in a forensic psychiatric patient sample, possibly highlighting a potential issue with these facet scales in the forensic psychiatric context.

Confirmatory factor analyses showed poor to mediocre fit for all three models, in essence replicating recent findings from Soe-Agnie et al. [34], carried out in a Dutch forensic psychiatric sample. Nevertheless, the bifactor model for the ESI-BF, first proposed and tested by Patrick et al. [1], showed the best fit out of the three models examined in terms of relative and absolute fit indices. Although absolute fit for the bifactor model was mediocre in our study, it is worth noting that its relative fit was similar to what has been observed in previous work [1, 34, 43]. Whether this can be seen as a vindication of the original models is difficult to tell given that bifactor models are known to be prone to overfitting [72]. It should also be noted that while it is theoretically possible to improve overall goodness-of-fit by post-hoc model modification based on modification indices, this approach remains debated due to issues with poor generalizability, especially in small samples [73–75]. Thus, given the relatively small sample in the current study, we chose not to pursue any post-hoc model modification. However, for the interested reader we provide all modification indices in the Supplemental Material (see supplementary material 6–8).

There are several possible explanations for the mediocre fit of the bifactor model in our study. First and foremost, the small sample size likely impacted the robustness of our analyses. Another reason could stem from a possible heterogeneity in the clinical manifestation of violence (e.g., psychotic, impulsive and organized violence; [76]) and other externalizing outcomes among our participants. It is an open question if violent and externalizing outcomes in our group was driven mainly by processes stemming from the severe mental illness (e.g., delusions) as opposed to personality traits along the externalizing spectrum, the two processes of which may contribute differentially to the manifestation of violence. The structural models tested in the current study were all originally derived by Patrick et al. [1] in undergraduate and correctional samples in which, presumably, the rate of severe mental illnesses was relatively low. Thus, it is possible that the high rates of severe mental illnesses and neurodevelopmental disorders that characterize our sample [49] contributed independently, beyond any externalizing psychopathology, to outcomes such as violence, and therefore in the end may have affected the fit of the models. Similarly, it is likely that the clinical characteristics of our sample differed from those of Soe-Agnie et al. [34], despite both being based on forensic psychiatric patients. This would be due to differences in the legislations and definitions pertaining to offenders with severe mental illnesses in Sweden and the Netherlands, resulting in more patients with a primary diagnosis of personality disorder as opposed to psychotic disorder in the Netherlands (for some estimates see: [50, 51, 77, 78].

With regards to the criterion validity measures, several robust correlations emerged. However, before interpreting these findings a significant caveat must be noted. As stated above, the correlated factors model used in this analysis exhibited mediocre model fit. Therefore, even though the observed correlations were robust, the relationship between the correlations and the structural fit of the models that is, how the structure and content of the ESI-BF give rise to these correlations remains unclear, and should be further investigated.

All ESI-BF subfactors were robustly associated with repeated truancy before the age of 18. This finding is in line with the notion that early and repeated truancy is best seen as a marker of a broad externalizing tendency and not only as a marker of low school engagement, although these processes may be reciprocal [79]. Early and repeated school truancy should thus warrant increased societal attention and intervention not only because of the risk for poor educational outcomes but also because it could portend a future trajectory of increasingly severe externalizing problems [79, 80].

In relation to the LHA, which to the best of our knowledge has not been studied in conjunction with either the ESI or ESI-BF before, our findings indicate that all three ESI-BF subfactors were positively and robustly associated with the LHA_TOT_, LHA_AGG_, and LHA_ANTI_ subscales. Thus, providing some support for the overarching convergent validity of the ESI-BF as the LHA indexes instances of violent, criminal and norm-breaking behavior. The strongest of these associations was for the ESI-BF_DIS_ subfactor, suggesting further support for the conception that disinhibition is a core aspect of externalizing psychopathology [12, 36, 81]. Of note is also that the only ESI-BF subfactor that exhibited a robust, positive, association with LHA_SELF_ was ESI-BF_DIS_. This finding may be interpreted in the light of previous studies that have pointed to a link between poor inhibitory control and impulsivity and non-suicidal self-injury [82, 83]. As the ESI-BF_DIS_ subfactor appears, to a relatively high degree, conceptually similar to those constructs, the association with LHA_SELF_ scores would seem to be theoretically buttressed by this literature.

Several specific associations for the ESI-BF_AGG_ subfactor emerged. It was the only subfactor to be robustly associated with violence towards a caregiver before the age of 18. Previous literature specifically examining the link between callous traits and violence towards caregivers appears scarce (for one recent example see Curtis et al. [84]). However, one model proposed by Kuay and colleagues [85] delineates two proposed groups of children who engage in child-to-parent aggression. One group, with children exhibiting a high degree of callous-unemotional traits who present with more proactive aggression and aggression extending beyond the family (denoted generalists). Another group, with low levels of callous-unemotional traits, do not typically display aggression towards other people than their parents and their aggression is primarily of a reactive character (denoted specialist). Interpreting these findings through the lens of this model may lend some support for the existence of Kuay and colleagues’ proposed generalist group and may also parallel the distinction between disinhibited and antagonistic externalizing in the HiTOP model [47]. A significant methodological limitation must however be accounted for here; our assessment of callous traits was done in adulthood and not in proximity with the violence towards the caregiver. It is thus not possible for us to establish the temporal precedence of these callous traits in the child-to-parent aggressors in our sample.

In line with some previous findings [33, 35], ESI-BF_AGG_ was also the subfactor that exhibited weaker associations with alcohol and substance abuse as compared to the other two subfactors. From the standpoint of previous research this appears unsurprising, as the ESI-BF_AGG_ was constructed to capture core aspects of interpersonal-affective processes of psychopathic personality traits, above and beyond the influence of the ESI-BF_DIS_. The ESI-BF_AGG_ has previously been labeled under the phenotypic concept of “Meanness” in Patrick and colleagues [86] triarchic conceptualization of psychopathy. Thus, it describes an individual with tendencies towards narcissism, callousness, proactive aggression and an antagonistic and disaffiliated interpersonal style. Such attributes are not necessarily connected to substance and alcohol abuse, which in fact to some degree often are social activities and as such would stand in contrast to the disaffiliated style of the individual with psychopathic traits. These results therefore also lie in line with the current HiTOP conceptualization of the externalizing spectrum which distinguishes between a disinhibited and an antagonistic component of externalizing [47].

Moreover, scores on the ESI-BF_AGG_ were negatively associated with having committed sexual crimes. Previous research on the link between callous traits and sexual offences appears to support such a connection [87–90], although exceptions also exist in the literature on juvenile sexual offenders [91]. Moreover, recent research in a sample of young Swedish offenders found a negative association between aggression and sexual offences [92] and an older study of Dutch forensic patients also found significantly lower levels of self-reported hostile and aggressive behaviors among patients convicted of sexual offences as opposed to among patients convicted other violent offences [93]. Our finding may reflect the nature of the particular sexual offenders found in our sample, as the strength of the association between psychopathic traits and sexual crimes has been found to vary considerably with regard to the type of sexual offence (e.g., rape, extra-familial offences or mixed sexual offences; [90]). It should also be noted here, however, that our number of sexual offenders in our sample is small and that these findings may also reflect legal praxis surrounding mentally disordered offenders in Sweden. Individuals who have committed sexual offenses and who present with personality disorders in the absence of conditions such as psychosis or autism, have over the last decades increasingly been sentenced to prison rather than to FPC and only a small minority of patients within Swedish FPC are now sentenced for a sexual index offence (7% of male patients and 1% of female patients; [51]). The sexual violence committed by the patients in our sample may therefore have been driven more by the nature and symptoms of their severe mental illness rather than by processes related to externalizing psychopathology, paraphilias or personality disorders.

Lastly, the ESI-BF was able to discern those patients who committed a larger number of crimes and those who began engaging in criminal behaviors at an early age. This finding suggests that externalizing problems are indeed distributed along a spectrum of severity and that this is, as previously described [94, 95], manifested by an early initiation of, and persistence in, criminal behaviors. Crucially, studies have shown that a significant subgroup (~ 20%) of individuals who later develop a schizophrenia spectrum disorder display early and extensive externalizing behaviors which persist over the life-course and are often captured in childhood by the diagnoses of CD and ODD [96, 97]. Accurate assessment of externalizing behaviors thus seems to be of high priority for this subgroup of persons with severe mental illnesses. For example, a recent large-scale epidemiological study [98] found comorbid SUDs in persons with severe mental illnesses to increase the risk not only for violence perpetration but also for violent victimization.

### Summary and conclusion

The potential relevance of ESI-BF is suggested by the robust associations between the ESI-BF subfactors and the measures of criterion validity examined in this study, and further by the good reliability and internal-consistency values exhibited by the ESI-BF. The current study constitutes a step towards making the ESI-BF, and the HiTOP taxonomy under which it is now subsumed, relevant for use within forensic psychiatric settings. While this study alone is of course insufficient to establish the validity or the appropriateness of the clinical use of the ESI-BF in forensic psychiatric settings, it adds a piece of the puzzle which was outlined by Simms and colleagues [99] in which the need for instruments derived from the HiTOP taxonomy to be increasingly tested in clinical and forensic samples is highlighted. In the long term, improved measurement and understanding of the externalizing spectrum may aid in the development of novel treatments within settings such as forensic services, possibly with a broader transdiagnostic approach [100]. Such transdiagnostic approaches have historically mainly been focused on internalizing aspects of psychopathology but findings relevant to externalizing problems, among both child and adult populations, are beginning to emerge. Examples of potential transdiagnostic mechanisms that have been implicated in these studies and which may serve as future treatment targets include: impulsivity, emotion dysregulation, irritability, anger and anger rumination and impaired emotion recognition [101–105]. Identifying and targeting such potential mechanism that cut across diagnostic categories and intersect with severe mental illness in this group of offenders could then potentially increase treatment efficacy. At least two such factors, anger and impulsivity, have already been highlighted as potentially relevant in relation to schizophrenia and its relationship with violence [106, 107].

### Strengths and limitations

A limitation in the present study was the relatively small sample size. This is common within forensic psychiatric patient samples but, of course, affects the robustness of the statistical analyses and, subsequently, any conclusions drawn from this study must take the small sample size into account. Another limitation relates to the accuracy of self-report measures in research on forensic psychiatric patients, a population with a high prevalence of psychotic disorders where reduced illness insight and impaired cognitive functions are common features [37]. Concerns may therefore be raised regarding to which degree the participants were willing and/or able to answer the questions of the ESI-BF truthfully and accurately. The context of compulsory FPC could also quite plausibly have affected the way our participants chose to present themselves on the questionnaire, although they were thoroughly informed about the absolute disconnect between their research participation and any decisions about their care. Although we have no formal way to test to which degree or in which direction, if any, such factors may have impacted the results, there is some literature suggesting that concerns of this nature may be unwarranted. Self-report measures in forensic populations may, overall, remain valid and patients with psychotic disorders may nonetheless be able to report accurately on aspect of themselves [108–111].

A major limitation in regard to our analyses of the criterion validity of the ESI-BF was the mediocre fit of the structural model we chose to use for those analyses. Such an analysis is in the light of this fact necessarily speculative and all results must be interpreted in the light of this significant limitation. Nonetheless, as this model has been used frequently in the ESI literature, we deemed it worth including.

Another limitation pertains to the use of cut-offs, both for the evaluation of CFA models and for interpreting Bayesian findings. While it may be argued that using cut-offs is arbitrary [66], thus prompting careful interpretation, they may nevertheless be heuristically useful.

A further potential limitation lies in the degree to which these findings may generalize to other forensic psychiatric populations. This is because the Swedish legislation regulating compulsory FPC is, with very few exceptions, internationally unique in its design [112]. This may hamper the generalizability of our results in other jurisdictions. Nonetheless, the group of offenders with severe mental illnesses studied here is of course not a group of persons unique to Sweden but one that would likely be found in similar systems and context in other nations and jurisdictions. This therefore suggest that some degree of generalizability outside of the Swedish FPC context, despite the legal peculiarities of Sweden, is present in the current study.

A strength of our current study is the rich, comprehensive array of criterion validity measures related to historic externalizing behaviors and outcomes for our participants. Given the extensive coverage of these information sources, and the combination of self-report and external sources, we believe that both these factors strengthen the reliability and accuracy of our data. With our access to this data, we could not only examine the structural aspects of the ESI-BF in this population but also gain a better picture of the validity of the ESI-BF and how it relates to “real world” outcomes that are thought to fall within the externalizing spectrum. Another strength, given the small sample size, is Bayesian approach to investigating criterion validity; Bayesian inferences remain valid regardless of sample size, and allows for genuine probabilistic statements [113].

### Clinical implications and future directions

We suggest that this study may aid not only researchers studying externalizing behaviors in forensic populations but that it may also, in the long run, contribute to the clinical assessment of these problems and behaviors in forensic psychiatric settings. Mounting evidence, which conforms with our clinical experience, points to the independent and primary importance of assessing and treating antisocial and externalizing behaviors in the prevention of violence and subsequent recidivism of offenders with severe mental illnesses [26–29, 114].

Furthermore, future research on the ESI-BF in forensic psychiatric context should seek to recruit larger samples in order to ascertain if this affects the model fit for Patrick’s original models [1], and in lieu of those models achieving acceptable fit further research could seek test the three-factor model that emerged in the work of Soe-Agnie et al. [34]. In a sample of Dutch forensic psychiatric patients, Soe-Agnie et al. [34] described a three-dimensional model, obtained via minimum rank factor analysis and exploratory bifactor analysis. The three dimensions were labeled Disinhibition/Alcohol Abuse, Callous Aggression and Drug Abuse. The Disinhibition/Alcohol Abuse factor contained facet scales tapping alcohol misuse and impulsive and irresponsible behaviors while the Drug Abuse factor encompassed facet scales related to drug abuse, physical aggression as well as other forms of antisocial behavior. Lastly, the Callous Aggression factor in Soe-Agnie’s model corresponded to the Callous Aggression factor found by Patrick et al. [1], with the exception of one facet scale, Physical Aggression [34].

Finally, since the ESI-BF was developed in a context where severe mental illness was not the primary clinical concern it is important that future investigations among forensic psychiatric patients continue to scrutinize if the original models hold up in these populations where externalizing psychopathology and severe mental illness intersect, or if a new superior structure emerges.

## Electronic supplementary material

Below is the link to the electronic supplementary material.

Supplementary Material 1 Clinical and criminological variables

Supplementary Material 2 Descriptives General Factor

Supplementary Material 3 Descriptives Substance Abuse Factor

Supplementary Material 4 Descriptives Callous Aggression Factor

Supplementary Material 5 Bifactor Model Correlations

Supplementary Material 6 Modification indices of the unidimensional model

Supplementary Material 7 Modification indices of the correlated factors model

Supplementary Material 8 Modification indices of the bifactor model

## Data Availability

Raw data for this study cannot be made publicly available for ethical reasons, as public availability would compromise patient confidentiality and/or participant privacy. The data contains sensitive information, such as detailed descriptions of crimes, mental disorders, and illicit drug use, and details about forensic psychiatric treatment which could be used to identify individuals. The data is stored on secured hard drives. For questions about data availability please address correspondence to the first author at: johan.berlin.7665@med.lu.se.

## Abbreviations

ESI: Externalizing Spectrum Inventory
ESI-BF: Externalizing Spectrum Inventory-Brief Form
LHA: Life History of Aggression
CFA: Confirmatory factor analysis
FPC: Forensic psychiatric care
HiTOP: Hierarchical Taxonomy of Psychopathology
CD: Conduct Disorder
ODD: Oppositional Defiant Disorder
ADHD: Attention Deficit Hyperactivity Disorder
ASPD: Antisocial Personality Disorder
SUDs: Substance Use Disorders
AIC: Akaike information criterion
SABIC: Sample-size adjusted Bayesian information criterion
CFI: Comparative fit index
TLI: Tucker-Lewis index
RMSEA: Root mean square error of approximation
SRMR: Standardized root mean square residual
HDI: Highest density interval
SD: Standard deviation
