# Supplementary material for: Exploring the psychometric properties of the externalizing spectrum inventory-brief form in a Swedish forensic psychiatric inpatient sample
Source: BMC Psychiatry. 2023 Mar 21;23:184. doi: 10.1186/s12888-023-04609-y (PMC10031895; doi:10.1186/s12888-023-04609-y)
Supplement: Supplementary file 1 — Supplementary Material 1 Clinical and criminological variables [file 12888_2023_4609_MOESM1_ESM.docx]

**Supplementary Material 1** - **Description of clinical and criminological measur****es and variables**

Supplementary Material 1 – .docx, “Clinical and criminological variables”. This file includes a description of how the clinical and criminological variables were defined and computed.

Participants were asked to rate their frequency of school truancy on a three-point scale: “repeatedly/multiple times”, “once or two times” or “never”. A new binary variable labelled “Repeated truancy” was created, with those classified as “repeatedly/multiple times” coded as “yes” and all others as “no”, resulting in a total of 50 participants (65%) coded as “yes”.

Participants were also asked about their past engagement in the bullying of others. Options were again: “repeatedly/multiple times”, “once or two times” or “never”. A new binary variable labeled “Repeated bullying” was created, with those classified repeatedly/multiple times coded as “yes” and all others as “no”, resulting in a total of 17 participants (22%) coded as “yes”.

Participants were also asked if they had ever engaged in violence towards a caregiver before the age of eighteen. Response categories were: “repeatedly/multiple times”, “once or two times” or “never”. A new binary variable labelled “Violence against caregiver” was created, with those classified “repeatedly/multiple times” and “once or two times” coded as “yes” and all others as “no”, resulting in a total of 33 participants (43%) coded as “yes”.

Finally, participants were asked about their alcohol and substance use habits. Alcohol use was coded on a three-point scale ranging from “never used”, “normal use” and “problematic use/abuse” as the categories. A new binary variable labelled “Alcohol use” was created, with those classified as, problematic use/abuse coded as “yes” and all other responses as “no”, resulting in a total of 45 participants (58%) coded as “yes”.

Substance use was coded in three categories: “repeated use”, “once or two times” and “never”. A new binary variable labelled “Substance use” was created, with those classified as, repeated use coded as “yes” and all other responses as “no”, resulting in a total of 59 participants (77%) coded as “yes”.

Information about previous criminal activity was obtained from sentencing records, previous files and assessment and through self-report and used to assess the degree to which participants had been involved in various types of crimes. Attempted crimes were also included in this variable.

Nine further binary variables pertaining to criminal activity were created as follows:

“Any lethal violence” was created, with participants that had self-reported and/or been sentenced for any type of crime involving lethal violence (e.g., murder, manslaughter) coded as “yes”, and all others as “no”, resulting in total of 20 participants (26%) coded as “yes”.

“Any assault including aggravated assault” was created, with participants that had self-reported and/or been sentenced for any type of crime involving an assault or aggravated assault coded as “yes”, and all others as “no”, resulting in total of 48 participants (62%) classified as “yes”.

“Other violent crimes” was created, with participants that had self-reported and/or been sentenced for any other type of violent crime (e.g. arson, robbery, unlawful threat) coded as “yes” and all others as “no”, resulting in a total of 61 participants (79%) classified as “yes”.

“Sexual crimes” was created, with participants that had self-reported and/or been sentenced for any other type of sexual crime (e.g. rape, indecent exposure, sexual molestation) coded as “yes” and all others as “no”, resulting in a total of 10 participants (13%) classified as “yes”.

“Theft and property crimes” was created, with participants that had self-reported and/or been sentenced for any type of theft or property related crime (e.g. burglary, shoplifting) coded as “yes” and all others as “no”, resulting in a total of 52 participants (68%) classified as “yes”.

“Financial crimes” was created, with participants that had self-reported and/or been sentenced for any other type of financial crime (e.g. fraud, embezzlement) coded as “yes” and all others as “no”, resulting in a total of 22 participants (29%) classified as “yes”.

“Traffic crimes” was created with, with participants who that had self-reported and/or been sentenced for any other type of traffic crime (e.g. speeding, drunk driving) coded as “yes” and all others as “no”, resulting in a total of 53 participants (69%) classified as “yes”.

“Drug-related crimes” was created with, with participants who that had self-reported and/or been sentenced for any other type of drug-related crime (e.g. unlawful possession, drug trafficking) coded as “yes” and all others as “no”, resulting in a total of 59 participants (77%) classified as “yes”.

Finally “Weapon-related crimes” was created with, with participants who that had self-reported and/or been sentenced for any other type of weapon-related crime (e.g. unlawful possession of firearm) coded as “yes” and all others as “no”, resulting in a total of 29 participants (38%) classified as “yes”.
