# Supplementary material for: Exploring the psychometric properties of the externalizing spectrum inventory-brief form in a Swedish forensic psychiatric inpatient sample
Source: BMC Psychiatry. 2023 Mar 21;23:184. doi: 10.1186/s12888-023-04609-y (PMC10031895; doi:10.1186/s12888-023-04609-y)
Supplement: Supplementary file 2 — Supplementary Material 2 Descriptives General Factor [file 12888_2023_4609_MOESM2_ESM.docx]

**Supplementary Material 2 – Descriptives General Factor**.

Supplementary Material 2 – .docx, “Descriptives General Factor”. This file includes results using the facet-based bifactor model specification.

Descriptive statistics (means and standard deviations) and posterior medians of the estimated difference for the General factor (λ_G_) of the bifactor model (N = 77).

| Measure | *M*_yes_ (SD) | *M*_no_ (SD) | Est. diff. [90 % HDI] |
| --- | --- | --- | --- |
| Repeated truancy | 0.23 (1) | -0.42 (0.78) | **0.68 [0.32, 1.03]** |
| Repeated bullying | 0.05 (0.96) | -0.02 (0.99) | 0.07 [-0.39, 0.54] |
| Any violence against caregiver | 0.22 (1.07) | -0.17 (0.88) | **0.42 [0.03, 0.81]** |
| Excessive alcohol use | 0.39 (0.9) | -0.55 (0.8) | **0.95 [0.62, 1.29]** |
| Excessive substance use | 0.21 (0.98) | -0.68 (0.6) | **0.9 [0.56, 1.22]** |
| Any sentence for deadly violence | -0.05 (0.87) | 0.02 (1.02) | -0.05 [-0.45, 0.38] |
| Multiple sentences for assault | 0.09 (0.99) | -0.15 (0.96) | 0.28 [-0.1, 0.67] |
| Multiple sentences for other violence crimes | -0.02 (0.98) | 0.09 (1) | -0.1 [-0.59, 0.38] |
| Any sentence for sexual crimes^1^ | -0.26 (0.79) | 0.03 (1.01) | -0.3 [-0.8, 0.2] |
| Multiple sentences for theft or damage to property | 0.16 (0.95) | -0.34 (0.96) | **0.55 [0.16, 0.96]** |
| Any sentence for economics-related crimes | 0.35 (0.89) | -0.14 (0.98) | **0.52 [0.12, 0.91]** |
| Any sentence for traffic-related crimes | 0.09 (1) | -0.21 (0.9) | 0.35 [-0.04, 0.74] |
| Multiple sentences for narcotics-related crimes | 0.23 (0.96) | -0.77 (0.53) | **1.01 [0.7, 1.31]** |
| Multiple sentences for weapons-related crimes | 0.25 (0.86) | -0.15 (1.02) | **0.45 [0.07, 0.82]** |

Note.^1^ N = 76 for sexual crimes. HDI, highest density interval. Estimated differences for which the 90% HDI does not contain zero are highlighted in bold.
