# Supplementary material for: Exploring the psychometric properties of the externalizing spectrum inventory-brief form in a Swedish forensic psychiatric inpatient sample
Source: BMC Psychiatry. 2023 Mar 21;23:184. doi: 10.1186/s12888-023-04609-y (PMC10031895; doi:10.1186/s12888-023-04609-y)
Supplement: Supplementary file 3 — Supplementary Material 3 Descriptives Substance Abuse Factor [file 12888_2023_4609_MOESM3_ESM.docx]

**Supplementary Material 3 – Descriptives Substance Abuse Factor**

Supplementary Material 3 – .docx, “Descriptives Substance Abuse Factor”. This file includes results using the facet-based bifactor model specification.

Descriptive statistics (means and standard deviations) and posterior medians of the estimated difference for the residual substance use factor (λ_RSUB_) of the bifactor model (N = 77).

| Measure | *M*_yes_ (SD) | *M*_no_ (SD) | Est. diff. [90 % HDI] |
| --- | --- | --- | --- |
| Repeated truancy | 0.01 (0.91) | -0.01 (0.77) | 0 [-0.32, 0.31] |
| Repeated bullying | 0.45 (0.91) | -0.13 (0.81) | **0.52 [0.16, 0.9]** |
| Any violence against caregiver | 0.24 (0.84) | -0.18 (0.83) | **0.44 [0.13, 0.74]** |
| Excessive alcohol use | -0.17 (0.8) | 0.24 (0.89) | **-0.38 [-0.7, -0.07]** |
| Excessive substance use | -0.03 (0.82) | 0.1 (1) | -0.1 [-0.51, 0.33] |
| Any sentence for deadly violence | 0.09 (0.63) | -0.03 (0.93) | 0.16 [-0.13, 0.45] |
| Multiple sentences for assault | 0.15 (0.82) | -0.24 (0.88) | **0.42 [0.12, 0.73]** |
| Multiple sentences for other violence crimes | 0.02 (0.89) | -0.06 (0.75) | 0.09 [-0.27, 0.45] |
| Any sentence for sexual crimes^1^ | -0.24 (0.61) | 0.03 (0.89) | -0.26 [-0.65, 0.16] |
| Multiple sentences for theft or damage to property | 0.02 (0.9) | -0.05 (0.78) | 0.06 [-0.27, 0.37] |
| Any sentence for economics-related crimes | 0.02 (0.89) | -0.01 (0.85) | 0.04 [-0.33, 0.38] |
| Any sentence for traffic-related crimes | 0 (0.81) | 0.01 (0.96) | 0.04 [-0.35, 0.42] |
| Multiple sentences for narcotics-related crimes | -0.02 (0.82) | 0.08 (1) | -0.07 [-0.49, 0.34] |
| Multiple sentences for weapons-related crimes | 0.13 (0.92) | -0.08 (0.82) | 0.21 [-0.13, 0.54] |

Note.^1^ N = 76 for sexual crimes. HDI, highest density interval. Estimated differences for which the 90% HDI does not contain zero are highlighted in bold.
