# Supplementary material for: Exploring the psychometric properties of the externalizing spectrum inventory-brief form in a Swedish forensic psychiatric inpatient sample
Source: BMC Psychiatry. 2023 Mar 21;23:184. doi: 10.1186/s12888-023-04609-y (PMC10031895; doi:10.1186/s12888-023-04609-y)
Supplement: Supplementary file 4 — Supplementary Material 4 Descriptives Callous Aggression Factor [file 12888_2023_4609_MOESM4_ESM.docx]

**Supplementary Material 4 – Descriptives Callous Aggression Factor**

Supplementary Material 4 – .docx, “Descriptives Callous Aggression Factor”. This file includes results using the facet-based bifactor model specification.

Descriptive statistics (means and standard deviations) and posterior medians of the estimated difference for the residual callous aggression factor (λ_RAGG_) of the bifactor model (N = 77).

| Measure | *M*_yes_ (SD) | *M*_no_ (SD) | Est. diff. [90 % HDI] |
| --- | --- | --- | --- |
| Repeated truancy | 0.15 (0.9) | -0.29 (1.03) | **0.48 [0.08, 0.89]** |
| Repeated bullying | -0.07 (1.06) | 0.02 (0.94) | -0.1 [-0.62, 0.4] |
| Any violence against caregiver | 0.04 (0.91) | -0.03 (1.01) | 0.09 [-0.28, 0.48] |
| Excessive alcohol use | 0.08 (0.88) | -0.12 (1.07) | 0.22 [-0.18, 0.62] |
| Excessive substance use | 0.31 (0.87) | -1.02 (0.34) | **1.36 [1.12, 1.6]** |
| Any sentence for deadly violence | 0.01 (1.1) | 0 (0.92) | 0 [-0.47, 0.49] |
| Multiple sentences for assault | 0.11 (0.96) | -0.18 (0.96) | 0.32 [-0.06, 0.71] |
| Multiple sentences for other violence crimes | 0.01 (0.96) | -0.03 (1.01) | 0.04 [-0.46, 0.53] |
| Any sentence for sexual crimes^1^ | -0.36 (1.18) | 0.04 (0.92) | -0.45 [-1.15, 0.28] |
| Multiple sentences for theft or damage to property | 0.1 (1.03) | -0.22 (0.79) | 0.33 [-0.04, 0.7] |
| Any sentence for economics-related crimes | 0.21 (0.88) | -0.08 (0.99) | 0.31 [-0.07, 0.72] |
| Any sentence for traffic-related crimes | 0.19 (0.93) | -0.41 (0.92) | **0.63 [0.22, 1.01]** |
| Multiple sentences for narcotics-related crimes | 0.29 (0.88) | -0.95 (0.52) | **1.31 [1.03, 1.58]** |
| Multiple sentences for weapons-related crimes | 0.19 (0.93) | -0.12 (0.98) | 0.33 [-0.05, 0.71] |

Note.^1^ N = 76 for sexual crimes. HDI, highest density interval. Estimated differences for which the 90% HDI does not contain zero are highlighted in bold.
