# Supplementary material for: Exploring the psychometric properties of the externalizing spectrum inventory-brief form in a Swedish forensic psychiatric inpatient sample
Source: BMC Psychiatry. 2023 Mar 21;23:184. doi: 10.1186/s12888-023-04609-y (PMC10031895; doi:10.1186/s12888-023-04609-y)
Supplement: Supplementary file 5 — Supplementary Material 5 Bifactor Model Correlations [file 12888_2023_4609_MOESM5_ESM.docx]

**Supplementary Material 5 – Bifactor Model Correlations**

Supplementary Material 5 – .docx, “Bifactor Model Correlations”. This file includes results using the facet-based bifactor model specification.

Posterior medians of the estimated correlation with factor scores from the bifactor model for Life History of Aggression and criminological measures.

|  |  | Est. corr. [90% HDI] | | |
| --- | --- | --- | --- | --- |
| Measure | *N* | λ_G_ | λ_RAGG_ | λ_RSUB_ |
| LHA_TOT_ | 75 | **.61 [.48, .73]** | .03 [-.16, .23] | -.01 [-.20, .18] |
| LHA_AGG_ | 77 | **.49 [.34, .63]** | .14 [-.05, .32] | -.04 [-.23, .16] |
| LHA_ANTI_ | 76 | **.61 [.48, .72]** | .03 [-.16, .23] | .12 [-.07, .31] |
| LHA_SELF_ | 75 | .14 [-.05, .33] | -.19 [-.38, .00] | -.16 [-.35, .03] |
| Age at first crime | 73 | **-.23 [-.43, -.03]** | -.10 [-.31, .11] | -.10 [-.30, .10] |
| Age at first sentence | 77 | **-.27 [-.45, -.09]** | -.10 [-.29, .09] | -.20 [-.38, -.02] |
| Total number of sentences | 77 | **.23 [.03, .43]** | .04 [-.17, .25] | .15 [-.06, .34] |
| Total number of prison sentences | 77 | .15 [-.06, .35] | .02 [-.19, .22] | .14 [-.06, .35] |

Note. λ_G_, the general factor of the bifactor; λ_RAGG_, the callous aggressive residual factor of the bifactor model; λ_RSUB_; the substance use residual factor of the bifactor model; HDI, highest density interval. Estimated correlations for which the 90% HDI does not contain zero are highlighted in bold.
