# Supplementary material for: Exploring the psychometric properties of the externalizing spectrum inventory-brief form in a Swedish forensic psychiatric inpatient sample
Source: BMC Psychiatry. 2023 Mar 21;23:184. doi: 10.1186/s12888-023-04609-y (PMC10031895; doi:10.1186/s12888-023-04609-y)
Supplement: Supplementary file 6 — Supplementary Material 6 Modification indices of the unidimensional model [file 12888_2023_4609_MOESM6_ESM.docx]

**Supplementary Material 6 - Modification indices of the unidimensional model.**

| Left hand | Operator | Right hand | Modification index |
| --- | --- | --- | --- |
| esi_problematic_impulsivity | ~~ | esi_irresponsibility | 1.58 |
| esi_problematic_impulsivity | ~~ | esi_theft | 0.77 |
| esi_problematic_impulsivity | ~~ | esi_fraud | 0.50 |
| esi_problematic_impulsivity | ~~ | esi_impatient_urgency | 8.53 |
| esi_problematic_impulsivity | ~~ | esi_planful_control | 26.48 |
| esi_problematic_impulsivity | ~~ | esi_dependability | 2.30 |
| esi_problematic_impulsivity | ~~ | esi_alienation | 9.19 |
| esi_problematic_impulsivity | ~~ | esi_boredom_proneness | 0.77 |
| esi_problematic_impulsivity | ~~ | esi_blame_externalization | 3.93 |
| esi_problematic_impulsivity | ~~ | esi_honesty | 0.09 |
| esi_problematic_impulsivity | ~~ | esi_rebelliousness | 0.15 |
| esi_problematic_impulsivity | ~~ | esi_physical_aggression | 0.00 |
| esi_problematic_impulsivity | ~~ | esi_destructive_aggression | 0.04 |
| esi_problematic_impulsivity | ~~ | esi_relational_aggression | 0.55 |
| esi_problematic_impulsivity | ~~ | esi_empathy | 1.96 |
| esi_problematic_impulsivity | ~~ | esi_excitement_seeking | 0.07 |
| esi_problematic_impulsivity | ~~ | esi_marijuana_use | 10.18 |
| esi_problematic_impulsivity | ~~ | esi_marijuana_problems | 3.06 |
| esi_problematic_impulsivity | ~~ | esi_drug_use | 15.11 |
| esi_problematic_impulsivity | ~~ | esi_drug_problems | 4.58 |
| esi_problematic_impulsivity | ~~ | esi_alcohol_use | 2.96 |
| esi_problematic_impulsivity | ~~ | esi_alcohol_problems | 0.18 |
| esi_irresponsibility | ~~ | esi_theft | 0.05 |
| esi_irresponsibility | ~~ | esi_fraud | 3.69 |
| esi_irresponsibility | ~~ | esi_impatient_urgency | 0.08 |
| esi_irresponsibility | ~~ | esi_planful_control | 0.01 |
| esi_irresponsibility | ~~ | esi_dependability | 1.74 |
| esi_irresponsibility | ~~ | esi_alienation | 5.83 |
| esi_irresponsibility | ~~ | esi_boredom_proneness | 1.45 |
| esi_irresponsibility | ~~ | esi_blame_externalization | 1.16 |
| esi_irresponsibility | ~~ | esi_honesty | 0.35 |
| esi_irresponsibility | ~~ | esi_rebelliousness | 0.10 |
| esi_irresponsibility | ~~ | esi_physical_aggression | 8.02 |
| esi_irresponsibility | ~~ | esi_destructive_aggression | 0.33 |
| esi_irresponsibility | ~~ | esi_relational_aggression | 0.17 |
| esi_irresponsibility | ~~ | esi_empathy | 4.40 |
| esi_irresponsibility | ~~ | esi_excitement_seeking | 1.54 |
| esi_irresponsibility | ~~ | esi_marijuana_use | 0.00 |
| esi_irresponsibility | ~~ | esi_marijuana_problems | 0.19 |
| esi_irresponsibility | ~~ | esi_drug_use | 0.01 |
| esi_irresponsibility | ~~ | esi_drug_problems | 0.21 |
| esi_irresponsibility | ~~ | esi_alcohol_use | 1.39 |
| esi_irresponsibility | ~~ | esi_alcohol_problems | 1.85 |
| esi_theft | ~~ | esi_fraud | 2.73 |
| esi_theft | ~~ | esi_impatient_urgency | 5.96 |
| esi_theft | ~~ | esi_planful_control | 1.26 |
| esi_theft | ~~ | esi_dependability | 2.16 |
| esi_theft | ~~ | esi_alienation | 1.80 |
| esi_theft | ~~ | esi_boredom_proneness | 1.66 |
| esi_theft | ~~ | esi_blame_externalization | 0.00 |
| esi_theft | ~~ | esi_honesty | 0.00 |
| esi_theft | ~~ | esi_rebelliousness | 0.66 |
| esi_theft | ~~ | esi_physical_aggression | 0.51 |
| esi_theft | ~~ | esi_destructive_aggression | 2.85 |
| esi_theft | ~~ | esi_relational_aggression | 0.26 |
| esi_theft | ~~ | esi_empathy | 3.00 |
| esi_theft | ~~ | esi_excitement_seeking | 0.60 |
| esi_theft | ~~ | esi_marijuana_use | 2.56 |
| esi_theft | ~~ | esi_marijuana_problems | 0.90 |
| esi_theft | ~~ | esi_drug_use | 0.57 |
| esi_theft | ~~ | esi_drug_problems | 2.05 |
| esi_theft | ~~ | esi_alcohol_use | 0.00 |
| esi_theft | ~~ | esi_alcohol_problems | 0.38 |
| esi_fraud | ~~ | esi_impatient_urgency | 0.24 |
| esi_fraud | ~~ | esi_planful_control | 1.69 |
| esi_fraud | ~~ | esi_dependability | 3.85 |
| esi_fraud | ~~ | esi_alienation | 1.00 |
| esi_fraud | ~~ | esi_boredom_proneness | 1.90 |
| esi_fraud | ~~ | esi_blame_externalization | 1.45 |
| esi_fraud | ~~ | esi_honesty | 3.12 |
| esi_fraud | ~~ | esi_rebelliousness | 1.20 |
| esi_fraud | ~~ | esi_physical_aggression | 0.47 |
| esi_fraud | ~~ | esi_destructive_aggression | 8.29 |
| esi_fraud | ~~ | esi_relational_aggression | 10.33 |
| esi_fraud | ~~ | esi_empathy | 1.03 |
| esi_fraud | ~~ | esi_excitement_seeking | 0.09 |
| esi_fraud | ~~ | esi_marijuana_use | 4.60 |
| esi_fraud | ~~ | esi_marijuana_problems | 1.65 |
| esi_fraud | ~~ | esi_drug_use | 3.63 |
| esi_fraud | ~~ | esi_drug_problems | 0.81 |
| esi_fraud | ~~ | esi_alcohol_use | 0.65 |
| esi_fraud | ~~ | esi_alcohol_problems | 0.04 |
| esi_impatient_urgency | ~~ | esi_planful_control | 4.62 |
| esi_impatient_urgency | ~~ | esi_dependability | 0.65 |
| esi_impatient_urgency | ~~ | esi_alienation | 0.25 |
| esi_impatient_urgency | ~~ | esi_boredom_proneness | 5.64 |
| esi_impatient_urgency | ~~ | esi_blame_externalization | 0.57 |
| esi_impatient_urgency | ~~ | esi_honesty | 0.61 |
| esi_impatient_urgency | ~~ | esi_rebelliousness | 0.05 |
| esi_impatient_urgency | ~~ | esi_physical_aggression | 0.89 |
| esi_impatient_urgency | ~~ | esi_destructive_aggression | 0.39 |
| esi_impatient_urgency | ~~ | esi_relational_aggression | 3.73 |
| esi_impatient_urgency | ~~ | esi_empathy | 0.55 |
| esi_impatient_urgency | ~~ | esi_excitement_seeking | 6.74 |
| esi_impatient_urgency | ~~ | esi_marijuana_use | 9.02 |
| esi_impatient_urgency | ~~ | esi_marijuana_problems | 9.70 |
| esi_impatient_urgency | ~~ | esi_drug_use | 3.89 |
| esi_impatient_urgency | ~~ | esi_drug_problems | 2.99 |
| esi_impatient_urgency | ~~ | esi_alcohol_use | 1.35 |
| esi_impatient_urgency | ~~ | esi_alcohol_problems | 0.03 |
| esi_planful_control | ~~ | esi_dependability | 6.65 |
| esi_planful_control | ~~ | esi_alienation | 1.82 |
| esi_planful_control | ~~ | esi_boredom_proneness | 0.92 |
| esi_planful_control | ~~ | esi_blame_externalization | 0.05 |
| esi_planful_control | ~~ | esi_honesty | 3.80 |
| esi_planful_control | ~~ | esi_rebelliousness | 0.29 |
| esi_planful_control | ~~ | esi_physical_aggression | 0.28 |
| esi_planful_control | ~~ | esi_destructive_aggression | 0.35 |
| esi_planful_control | ~~ | esi_relational_aggression | 0.50 |
| esi_planful_control | ~~ | esi_empathy | 0.02 |
| esi_planful_control | ~~ | esi_excitement_seeking | 3.24 |
| esi_planful_control | ~~ | esi_marijuana_use | 4.52 |
| esi_planful_control | ~~ | esi_marijuana_problems | 2.36 |
| esi_planful_control | ~~ | esi_drug_use | 6.53 |
| esi_planful_control | ~~ | esi_drug_problems | 5.35 |
| esi_planful_control | ~~ | esi_alcohol_use | 0.82 |
| esi_planful_control | ~~ | esi_alcohol_problems | 0.04 |
| esi_dependability | ~~ | esi_alienation | 0.66 |
| esi_dependability | ~~ | esi_boredom_proneness | 0.25 |
| esi_dependability | ~~ | esi_blame_externalization | 2.12 |
| esi_dependability | ~~ | esi_honesty | 11.24 |
| esi_dependability | ~~ | esi_rebelliousness | 1.43 |
| esi_dependability | ~~ | esi_physical_aggression | 0.00 |
| esi_dependability | ~~ | esi_destructive_aggression | 0.04 |
| esi_dependability | ~~ | esi_relational_aggression | 0.00 |
| esi_dependability | ~~ | esi_empathy | 0.50 |
| esi_dependability | ~~ | esi_excitement_seeking | 0.24 |
| esi_dependability | ~~ | esi_marijuana_use | 3.87 |
| esi_dependability | ~~ | esi_marijuana_problems | 0.05 |
| esi_dependability | ~~ | esi_drug_use | 7.40 |
| esi_dependability | ~~ | esi_drug_problems | 3.70 |
| esi_dependability | ~~ | esi_alcohol_use | 0.40 |
| esi_dependability | ~~ | esi_alcohol_problems | 4.52 |
| esi_alienation | ~~ | esi_boredom_proneness | 0.09 |
| esi_alienation | ~~ | esi_blame_externalization | 12.18 |
| esi_alienation | ~~ | esi_honesty | 3.99 |
| esi_alienation | ~~ | esi_rebelliousness | 0.39 |
| esi_alienation | ~~ | esi_physical_aggression | 2.08 |
| esi_alienation | ~~ | esi_destructive_aggression | 0.14 |
| esi_alienation | ~~ | esi_relational_aggression | 0.03 |
| esi_alienation | ~~ | esi_empathy | 3.08 |
| esi_alienation | ~~ | esi_excitement_seeking | 3.82 |
| esi_alienation | ~~ | esi_marijuana_use | 0.40 |
| esi_alienation | ~~ | esi_marijuana_problems | 0.25 |
| esi_alienation | ~~ | esi_drug_use | 0.01 |
| esi_alienation | ~~ | esi_drug_problems | 0.63 |
| esi_alienation | ~~ | esi_alcohol_use | 0.07 |
| esi_alienation | ~~ | esi_alcohol_problems | 2.33 |
| esi_boredom_proneness | ~~ | esi_blame_externalization | 0.84 |
| esi_boredom_proneness | ~~ | esi_honesty | 2.24 |
| esi_boredom_proneness | ~~ | esi_rebelliousness | 0.25 |
| esi_boredom_proneness | ~~ | esi_physical_aggression | 0.02 |
| esi_boredom_proneness | ~~ | esi_destructive_aggression | 0.34 |
| esi_boredom_proneness | ~~ | esi_relational_aggression | 1.07 |
| esi_boredom_proneness | ~~ | esi_empathy | 0.91 |
| esi_boredom_proneness | ~~ | esi_excitement_seeking | 5.39 |
| esi_boredom_proneness | ~~ | esi_marijuana_use | 0.13 |
| esi_boredom_proneness | ~~ | esi_marijuana_problems | 0.78 |
| esi_boredom_proneness | ~~ | esi_drug_use | 0.01 |
| esi_boredom_proneness | ~~ | esi_drug_problems | 0.29 |
| esi_boredom_proneness | ~~ | esi_alcohol_use | 0.98 |
| esi_boredom_proneness | ~~ | esi_alcohol_problems | 0.02 |
| esi_blame_externalization | ~~ | esi_honesty | 6.36 |
| esi_blame_externalization | ~~ | esi_rebelliousness | 0.00 |
| esi_blame_externalization | ~~ | esi_physical_aggression | 0.08 |
| esi_blame_externalization | ~~ | esi_destructive_aggression | 1.91 |
| esi_blame_externalization | ~~ | esi_relational_aggression | 0.41 |
| esi_blame_externalization | ~~ | esi_empathy | 4.09 |
| esi_blame_externalization | ~~ | esi_excitement_seeking | 0.75 |
| esi_blame_externalization | ~~ | esi_marijuana_use | 0.01 |
| esi_blame_externalization | ~~ | esi_marijuana_problems | 0.05 |
| esi_blame_externalization | ~~ | esi_drug_use | 0.20 |
| esi_blame_externalization | ~~ | esi_drug_problems | 0.55 |
| esi_blame_externalization | ~~ | esi_alcohol_use | 0.05 |
| esi_blame_externalization | ~~ | esi_alcohol_problems | 1.14 |
| esi_honesty | ~~ | esi_rebelliousness | 0.41 |
| esi_honesty | ~~ | esi_physical_aggression | 0.12 |
| esi_honesty | ~~ | esi_destructive_aggression | 0.00 |
| esi_honesty | ~~ | esi_relational_aggression | 3.70 |
| esi_honesty | ~~ | esi_empathy | 7.34 |
| esi_honesty | ~~ | esi_excitement_seeking | 2.87 |
| esi_honesty | ~~ | esi_marijuana_use | 2.03 |
| esi_honesty | ~~ | esi_marijuana_problems | 4.12 |
| esi_honesty | ~~ | esi_drug_use | 5.14 |
| esi_honesty | ~~ | esi_drug_problems | 1.83 |
| esi_honesty | ~~ | esi_alcohol_use | 2.90 |
| esi_honesty | ~~ | esi_alcohol_problems | 0.03 |
| esi_rebelliousness | ~~ | esi_physical_aggression | 0.25 |
| esi_rebelliousness | ~~ | esi_destructive_aggression | 0.13 |
| esi_rebelliousness | ~~ | esi_relational_aggression | 2.23 |
| esi_rebelliousness | ~~ | esi_empathy | 0.33 |
| esi_rebelliousness | ~~ | esi_excitement_seeking | 1.68 |
| esi_rebelliousness | ~~ | esi_marijuana_use | 0.04 |
| esi_rebelliousness | ~~ | esi_marijuana_problems | 0.14 |
| esi_rebelliousness | ~~ | esi_drug_use | 0.97 |
| esi_rebelliousness | ~~ | esi_drug_problems | 2.94 |
| esi_rebelliousness | ~~ | esi_alcohol_use | 0.43 |
| esi_rebelliousness | ~~ | esi_alcohol_problems | 0.42 |
| esi_physical_aggression | ~~ | esi_destructive_aggression | 0.92 |
| esi_physical_aggression | ~~ | esi_relational_aggression | 5.27 |
| esi_physical_aggression | ~~ | esi_empathy | 10.30 |
| esi_physical_aggression | ~~ | esi_excitement_seeking | 1.68 |
| esi_physical_aggression | ~~ | esi_marijuana_use | 0.38 |
| esi_physical_aggression | ~~ | esi_marijuana_problems | 0.23 |
| esi_physical_aggression | ~~ | esi_drug_use | 0.51 |
| esi_physical_aggression | ~~ | esi_drug_problems | 0.96 |
| esi_physical_aggression | ~~ | esi_alcohol_use | 0.82 |
| esi_physical_aggression | ~~ | esi_alcohol_problems | 1.37 |
| esi_destructive_aggression | ~~ | esi_relational_aggression | 7.62 |
| esi_destructive_aggression | ~~ | esi_empathy | 2.44 |
| esi_destructive_aggression | ~~ | esi_excitement_seeking | 0.01 |
| esi_destructive_aggression | ~~ | esi_marijuana_use | 9.07 |
| esi_destructive_aggression | ~~ | esi_marijuana_problems | 5.90 |
| esi_destructive_aggression | ~~ | esi_drug_use | 4.38 |
| esi_destructive_aggression | ~~ | esi_drug_problems | 1.54 |
| esi_destructive_aggression | ~~ | esi_alcohol_use | 0.35 |
| esi_destructive_aggression | ~~ | esi_alcohol_problems | 1.63 |
| esi_relational_aggression | ~~ | esi_empathy | 12.84 |
| esi_relational_aggression | ~~ | esi_excitement_seeking | 0.04 |
| esi_relational_aggression | ~~ | esi_marijuana_use | 13.15 |
| esi_relational_aggression | ~~ | esi_marijuana_problems | 8.53 |
| esi_relational_aggression | ~~ | esi_drug_use | 8.15 |
| esi_relational_aggression | ~~ | esi_drug_problems | 4.80 |
| esi_relational_aggression | ~~ | esi_alcohol_use | 0.67 |
| esi_relational_aggression | ~~ | esi_alcohol_problems | 2.52 |
| esi_empathy | ~~ | esi_excitement_seeking | 5.63 |
| esi_empathy | ~~ | esi_marijuana_use | 4.02 |
| esi_empathy | ~~ | esi_marijuana_problems | 1.50 |
| esi_empathy | ~~ | esi_drug_use | 1.49 |
| esi_empathy | ~~ | esi_drug_problems | 0.20 |
| esi_empathy | ~~ | esi_alcohol_use | 0.01 |
| esi_empathy | ~~ | esi_alcohol_problems | 1.03 |
| esi_excitement_seeking | ~~ | esi_marijuana_use | 2.07 |
| esi_excitement_seeking | ~~ | esi_marijuana_problems | 2.84 |
| esi_excitement_seeking | ~~ | esi_drug_use | 0.13 |
| esi_excitement_seeking | ~~ | esi_drug_problems | 4.29 |
| esi_excitement_seeking | ~~ | esi_alcohol_use | 0.11 |
| esi_excitement_seeking | ~~ | esi_alcohol_problems | 4.92 |
| esi_marijuana_use | ~~ | esi_marijuana_problems | 38.04 |
| esi_marijuana_use | ~~ | esi_drug_use | 46.62 |
| esi_marijuana_use | ~~ | esi_drug_problems | 30.80 |
| esi_marijuana_use | ~~ | esi_alcohol_use | 0.06 |
| esi_marijuana_use | ~~ | esi_alcohol_problems | 0.14 |
| esi_marijuana_problems | ~~ | esi_drug_use | 15.94 |
| esi_marijuana_problems | ~~ | esi_drug_problems | 15.57 |
| esi_marijuana_problems | ~~ | esi_alcohol_use | 0.10 |
| esi_marijuana_problems | ~~ | esi_alcohol_problems | 0.00 |
| esi_drug_use | ~~ | esi_drug_problems | 30.42 |
| esi_drug_use | ~~ | esi_alcohol_use | 0.70 |
| esi_drug_use | ~~ | esi_alcohol_problems | 0.09 |
| esi_drug_problems | ~~ | esi_alcohol_use | 1.39 |
| esi_drug_problems | ~~ | esi_alcohol_problems | 0.96 |
| esi_alcohol_use | ~~ | esi_alcohol_problems | 15.61 |
