# Supplementary material for: Exploring the psychometric properties of the externalizing spectrum inventory-brief form in a Swedish forensic psychiatric inpatient sample
Source: BMC Psychiatry. 2023 Mar 21;23:184. doi: 10.1186/s12888-023-04609-y (PMC10031895; doi:10.1186/s12888-023-04609-y)
Supplement: Supplementary file 7 — Supplementary Material 7 Modification indices of the correlated factors model [file 12888_2023_4609_MOESM7_ESM.docx]

**Supplementary Material 7 - Modification indices of the correlated factors model.**

| Left hand | Operator | Right hand | Modification index |
| --- | --- | --- | --- |
| general_disinhibition | =~ | esi_honesty | 0.00 |
| general_disinhibition | =~ | esi_physical_aggression | 0.62 |
| general_disinhibition | =~ | esi_destructive_aggression | 2.35 |
| general_disinhibition | =~ | esi_relational_aggression | 0.61 |
| general_disinhibition | =~ | esi_empathy | 14.26 |
| general_disinhibition | =~ | esi_excitement_seeking | 7.49 |
| general_disinhibition | =~ | esi_marijuana_use | 14.40 |
| general_disinhibition | =~ | esi_marijuana_problems | 1.35 |
| general_disinhibition | =~ | esi_drug_use | 0.26 |
| general_disinhibition | =~ | esi_drug_problems | 15.28 |
| general_disinhibition | =~ | esi_alcohol_use | 1.50 |
| general_disinhibition | =~ | esi_alcohol_problems | 8.45 |
| callous_aggression | =~ | esi_problematic_impulsivity | 1.22 |
| callous_aggression | =~ | esi_irresponsibility | 7.28 |
| callous_aggression | =~ | esi_theft | 0.24 |
| callous_aggression | =~ | esi_fraud | 7.87 |
| callous_aggression | =~ | esi_impatient_urgency | 4.13 |
| callous_aggression | =~ | esi_planful_control | 0.05 |
| callous_aggression | =~ | esi_dependability | 0.18 |
| callous_aggression | =~ | esi_alienation | 4.04 |
| callous_aggression | =~ | esi_boredom_proneness | 0.45 |
| callous_aggression | =~ | esi_marijuana_use | 15.38 |
| callous_aggression | =~ | esi_marijuana_problems | 2.84 |
| callous_aggression | =~ | esi_drug_use | 0.00 |
| callous_aggression | =~ | esi_drug_problems | 16.02 |
| callous_aggression | =~ | esi_alcohol_use | 2.85 |
| callous_aggression | =~ | esi_alcohol_problems | 2.73 |
| substance_abuse | =~ | esi_problematic_impulsivity | 6.16 |
| substance_abuse | =~ | esi_irresponsibility | 4.37 |
| substance_abuse | =~ | esi_theft | 12.50 |
| substance_abuse | =~ | esi_fraud | 0.06 |
| substance_abuse | =~ | esi_impatient_urgency | 3.68 |
| substance_abuse | =~ | esi_planful_control | 3.71 |
| substance_abuse | =~ | esi_dependability | 1.30 |
| substance_abuse | =~ | esi_alienation | 0.17 |
| substance_abuse | =~ | esi_boredom_proneness | 1.62 |
| substance_abuse | =~ | esi_honesty | 0.72 |
| substance_abuse | =~ | esi_physical_aggression | 3.25 |
| substance_abuse | =~ | esi_destructive_aggression | 0.07 |
| substance_abuse | =~ | esi_relational_aggression | 3.16 |
| substance_abuse | =~ | esi_empathy | 1.97 |
| substance_abuse | =~ | esi_excitement_seeking | 3.63 |
| esi_problematic_impulsivity | ~~ | esi_irresponsibility | 0.02 |
| esi_problematic_impulsivity | ~~ | esi_theft | 1.73 |
| esi_problematic_impulsivity | ~~ | esi_fraud | 6.42 |
| esi_problematic_impulsivity | ~~ | esi_impatient_urgency | 4.48 |
| esi_problematic_impulsivity | ~~ | esi_planful_control | 22.26 |
| esi_problematic_impulsivity | ~~ | esi_dependability | 0.06 |
| esi_problematic_impulsivity | ~~ | esi_alienation | 8.26 |
| esi_problematic_impulsivity | ~~ | esi_boredom_proneness | 0.22 |
| esi_problematic_impulsivity | ~~ | esi_honesty | 1.01 |
| esi_problematic_impulsivity | ~~ | esi_physical_aggression | 0.08 |
| esi_problematic_impulsivity | ~~ | esi_destructive_aggression | 0.31 |
| esi_problematic_impulsivity | ~~ | esi_relational_aggression | 0.09 |
| esi_problematic_impulsivity | ~~ | esi_empathy | 3.85 |
| esi_problematic_impulsivity | ~~ | esi_excitement_seeking | 0.00 |
| esi_problematic_impulsivity | ~~ | esi_marijuana_use | 1.34 |
| esi_problematic_impulsivity | ~~ | esi_marijuana_problems | 0.10 |
| esi_problematic_impulsivity | ~~ | esi_drug_use | 4.14 |
| esi_problematic_impulsivity | ~~ | esi_drug_problems | 1.60 |
| esi_problematic_impulsivity | ~~ | esi_alcohol_use | 0.29 |
| esi_problematic_impulsivity | ~~ | esi_alcohol_problems | 1.26 |
| esi_irresponsibility | ~~ | esi_theft | 0.93 |
| esi_irresponsibility | ~~ | esi_fraud | 2.45 |
| esi_irresponsibility | ~~ | esi_impatient_urgency | 0.52 |
| esi_irresponsibility | ~~ | esi_planful_control | 2.53 |
| esi_irresponsibility | ~~ | esi_dependability | 0.20 |
| esi_irresponsibility | ~~ | esi_alienation | 5.53 |
| esi_irresponsibility | ~~ | esi_boredom_proneness | 1.60 |
| esi_irresponsibility | ~~ | esi_honesty | 0.36 |
| esi_irresponsibility | ~~ | esi_physical_aggression | 2.61 |
| esi_irresponsibility | ~~ | esi_destructive_aggression | 0.04 |
| esi_irresponsibility | ~~ | esi_relational_aggression | 0.03 |
| esi_irresponsibility | ~~ | esi_empathy | 3.56 |
| esi_irresponsibility | ~~ | esi_excitement_seeking | 0.02 |
| esi_irresponsibility | ~~ | esi_marijuana_use | 1.01 |
| esi_irresponsibility | ~~ | esi_marijuana_problems | 0.12 |
| esi_irresponsibility | ~~ | esi_drug_use | 1.28 |
| esi_irresponsibility | ~~ | esi_drug_problems | 0.70 |
| esi_irresponsibility | ~~ | esi_alcohol_use | 0.49 |
| esi_irresponsibility | ~~ | esi_alcohol_problems | 1.07 |
| esi_theft | ~~ | esi_fraud | 4.33 |
| esi_theft | ~~ | esi_impatient_urgency | 7.07 |
| esi_theft | ~~ | esi_planful_control | 2.66 |
| esi_theft | ~~ | esi_dependability | 2.74 |
| esi_theft | ~~ | esi_alienation | 1.06 |
| esi_theft | ~~ | esi_boredom_proneness | 0.38 |
| esi_theft | ~~ | esi_honesty | 0.00 |
| esi_theft | ~~ | esi_physical_aggression | 0.24 |
| esi_theft | ~~ | esi_destructive_aggression | 4.19 |
| esi_theft | ~~ | esi_relational_aggression | 0.06 |
| esi_theft | ~~ | esi_empathy | 2.32 |
| esi_theft | ~~ | esi_excitement_seeking | 0.02 |
| esi_theft | ~~ | esi_marijuana_use | 3.46 |
| esi_theft | ~~ | esi_marijuana_problems | 0.20 |
| esi_theft | ~~ | esi_drug_use | 0.02 |
| esi_theft | ~~ | esi_drug_problems | 0.14 |
| esi_theft | ~~ | esi_alcohol_use | 0.03 |
| esi_theft | ~~ | esi_alcohol_problems | 0.96 |
| esi_fraud | ~~ | esi_impatient_urgency | 2.40 |
| esi_fraud | ~~ | esi_planful_control | 8.33 |
| esi_fraud | ~~ | esi_dependability | 1.58 |
| esi_fraud | ~~ | esi_alienation | 2.00 |
| esi_fraud | ~~ | esi_boredom_proneness | 2.44 |
| esi_fraud | ~~ | esi_honesty | 0.95 |
| esi_fraud | ~~ | esi_physical_aggression | 1.47 |
| esi_fraud | ~~ | esi_destructive_aggression | 3.96 |
| esi_fraud | ~~ | esi_relational_aggression | 7.24 |
| esi_fraud | ~~ | esi_empathy | 0.32 |
| esi_fraud | ~~ | esi_excitement_seeking | 0.68 |
| esi_fraud | ~~ | esi_marijuana_use | 1.08 |
| esi_fraud | ~~ | esi_marijuana_problems | 0.03 |
| esi_fraud | ~~ | esi_drug_use | 0.01 |
| esi_fraud | ~~ | esi_drug_problems | 1.34 |
| esi_fraud | ~~ | esi_alcohol_use | 0.01 |
| esi_fraud | ~~ | esi_alcohol_problems | 0.01 |
| esi_impatient_urgency | ~~ | esi_planful_control | 1.73 |
| esi_impatient_urgency | ~~ | esi_dependability | 0.01 |
| esi_impatient_urgency | ~~ | esi_alienation | 0.99 |
| esi_impatient_urgency | ~~ | esi_boredom_proneness | 4.80 |
| esi_impatient_urgency | ~~ | esi_honesty | 0.00 |
| esi_impatient_urgency | ~~ | esi_physical_aggression | 0.15 |
| esi_impatient_urgency | ~~ | esi_destructive_aggression | 0.53 |
| esi_impatient_urgency | ~~ | esi_relational_aggression | 0.92 |
| esi_impatient_urgency | ~~ | esi_empathy | 0.04 |
| esi_impatient_urgency | ~~ | esi_excitement_seeking | 2.68 |
| esi_impatient_urgency | ~~ | esi_marijuana_use | 3.63 |
| esi_impatient_urgency | ~~ | esi_marijuana_problems | 4.79 |
| esi_impatient_urgency | ~~ | esi_drug_use | 0.76 |
| esi_impatient_urgency | ~~ | esi_drug_problems | 0.92 |
| esi_impatient_urgency | ~~ | esi_alcohol_use | 0.05 |
| esi_impatient_urgency | ~~ | esi_alcohol_problems | 0.51 |
| esi_planful_control | ~~ | esi_dependability | 3.09 |
| esi_planful_control | ~~ | esi_alienation | 0.78 |
| esi_planful_control | ~~ | esi_boredom_proneness | 0.28 |
| esi_planful_control | ~~ | esi_honesty | 2.74 |
| esi_planful_control | ~~ | esi_physical_aggression | 0.61 |
| esi_planful_control | ~~ | esi_destructive_aggression | 0.04 |
| esi_planful_control | ~~ | esi_relational_aggression | 4.01 |
| esi_planful_control | ~~ | esi_empathy | 0.01 |
| esi_planful_control | ~~ | esi_excitement_seeking | 2.03 |
| esi_planful_control | ~~ | esi_marijuana_use | 0.07 |
| esi_planful_control | ~~ | esi_marijuana_problems | 0.02 |
| esi_planful_control | ~~ | esi_drug_use | 0.57 |
| esi_planful_control | ~~ | esi_drug_problems | 0.12 |
| esi_planful_control | ~~ | esi_alcohol_use | 0.00 |
| esi_planful_control | ~~ | esi_alcohol_problems | 0.39 |
| esi_dependability | ~~ | esi_alienation | 1.87 |
| esi_dependability | ~~ | esi_boredom_proneness | 0.78 |
| esi_dependability | ~~ | esi_honesty | 10.45 |
| esi_dependability | ~~ | esi_physical_aggression | 0.02 |
| esi_dependability | ~~ | esi_destructive_aggression | 0.46 |
| esi_dependability | ~~ | esi_relational_aggression | 0.93 |
| esi_dependability | ~~ | esi_empathy | 0.32 |
| esi_dependability | ~~ | esi_excitement_seeking | 0.01 |
| esi_dependability | ~~ | esi_marijuana_use | 0.45 |
| esi_dependability | ~~ | esi_marijuana_problems | 3.95 |
| esi_dependability | ~~ | esi_drug_use | 3.67 |
| esi_dependability | ~~ | esi_drug_problems | 0.13 |
| esi_dependability | ~~ | esi_alcohol_use | 2.26 |
| esi_dependability | ~~ | esi_alcohol_problems | 6.08 |
| esi_alienation | ~~ | esi_boredom_proneness | 0.10 |
| esi_alienation | ~~ | esi_honesty | 3.88 |
| esi_alienation | ~~ | esi_physical_aggression | 0.39 |
| esi_alienation | ~~ | esi_destructive_aggression | 0.89 |
| esi_alienation | ~~ | esi_relational_aggression | 0.19 |
| esi_alienation | ~~ | esi_empathy | 2.41 |
| esi_alienation | ~~ | esi_excitement_seeking | 1.64 |
| esi_alienation | ~~ | esi_marijuana_use | 0.10 |
| esi_alienation | ~~ | esi_marijuana_problems | 0.07 |
| esi_alienation | ~~ | esi_drug_use | 0.70 |
| esi_alienation | ~~ | esi_drug_problems | 0.43 |
| esi_alienation | ~~ | esi_alcohol_use | 0.00 |
| esi_alienation | ~~ | esi_alcohol_problems | 2.09 |
| esi_boredom_proneness | ~~ | esi_honesty | 3.49 |
| esi_boredom_proneness | ~~ | esi_physical_aggression | 0.04 |
| esi_boredom_proneness | ~~ | esi_destructive_aggression | 0.17 |
| esi_boredom_proneness | ~~ | esi_relational_aggression | 2.48 |
| esi_boredom_proneness | ~~ | esi_empathy | 1.12 |
| esi_boredom_proneness | ~~ | esi_excitement_seeking | 6.69 |
| esi_boredom_proneness | ~~ | esi_marijuana_use | 0.17 |
| esi_boredom_proneness | ~~ | esi_marijuana_problems | 1.46 |
| esi_boredom_proneness | ~~ | esi_drug_use | 0.01 |
| esi_boredom_proneness | ~~ | esi_drug_problems | 0.43 |
| esi_boredom_proneness | ~~ | esi_alcohol_use | 0.50 |
| esi_boredom_proneness | ~~ | esi_alcohol_problems | 0.00 |
| esi_honesty | ~~ | esi_physical_aggression | 0.97 |
| esi_honesty | ~~ | esi_destructive_aggression | 2.33 |
| esi_honesty | ~~ | esi_relational_aggression | 0.18 |
| esi_honesty | ~~ | esi_empathy | 2.73 |
| esi_honesty | ~~ | esi_excitement_seeking | 0.39 |
| esi_honesty | ~~ | esi_marijuana_use | 0.35 |
| esi_honesty | ~~ | esi_marijuana_problems | 0.88 |
| esi_honesty | ~~ | esi_drug_use | 2.03 |
| esi_honesty | ~~ | esi_drug_problems | 0.02 |
| esi_honesty | ~~ | esi_alcohol_use | 4.84 |
| esi_honesty | ~~ | esi_alcohol_problems | 0.32 |
| esi_physical_aggression | ~~ | esi_destructive_aggression | 0.19 |
| esi_physical_aggression | ~~ | esi_relational_aggression | 0.43 |
| esi_physical_aggression | ~~ | esi_empathy | 3.20 |
| esi_physical_aggression | ~~ | esi_excitement_seeking | 0.20 |
| esi_physical_aggression | ~~ | esi_marijuana_use | 0.20 |
| esi_physical_aggression | ~~ | esi_marijuana_problems | 0.11 |
| esi_physical_aggression | ~~ | esi_drug_use | 0.38 |
| esi_physical_aggression | ~~ | esi_drug_problems | 2.32 |
| esi_physical_aggression | ~~ | esi_alcohol_use | 0.91 |
| esi_physical_aggression | ~~ | esi_alcohol_problems | 0.47 |
| esi_destructive_aggression | ~~ | esi_relational_aggression | 1.29 |
| esi_destructive_aggression | ~~ | esi_empathy | 0.26 |
| esi_destructive_aggression | ~~ | esi_excitement_seeking | 2.05 |
| esi_destructive_aggression | ~~ | esi_marijuana_use | 2.42 |
| esi_destructive_aggression | ~~ | esi_marijuana_problems | 0.64 |
| esi_destructive_aggression | ~~ | esi_drug_use | 0.07 |
| esi_destructive_aggression | ~~ | esi_drug_problems | 1.55 |
| esi_destructive_aggression | ~~ | esi_alcohol_use | 0.00 |
| esi_destructive_aggression | ~~ | esi_alcohol_problems | 0.01 |
| esi_relational_aggression | ~~ | esi_empathy | 3.63 |
| esi_relational_aggression | ~~ | esi_excitement_seeking | 6.74 |
| esi_relational_aggression | ~~ | esi_marijuana_use | 2.35 |
| esi_relational_aggression | ~~ | esi_marijuana_problems | 0.67 |
| esi_relational_aggression | ~~ | esi_drug_use | 0.02 |
| esi_relational_aggression | ~~ | esi_drug_problems | 0.79 |
| esi_relational_aggression | ~~ | esi_alcohol_use | 0.02 |
| esi_relational_aggression | ~~ | esi_alcohol_problems | 0.08 |
| esi_empathy | ~~ | esi_excitement_seeking | 0.17 |
| esi_empathy | ~~ | esi_marijuana_use | 1.07 |
| esi_empathy | ~~ | esi_marijuana_problems | 0.00 |
| esi_empathy | ~~ | esi_drug_use | 0.04 |
| esi_empathy | ~~ | esi_drug_problems | 0.65 |
| esi_empathy | ~~ | esi_alcohol_use | 0.07 |
| esi_empathy | ~~ | esi_alcohol_problems | 0.36 |
| esi_excitement_seeking | ~~ | esi_marijuana_use | 0.23 |
| esi_excitement_seeking | ~~ | esi_marijuana_problems | 0.16 |
| esi_excitement_seeking | ~~ | esi_drug_use | 5.09 |
| esi_excitement_seeking | ~~ | esi_drug_problems | 2.44 |
| esi_excitement_seeking | ~~ | esi_alcohol_use | 0.00 |
| esi_excitement_seeking | ~~ | esi_alcohol_problems | 1.38 |
| esi_marijuana_use | ~~ | esi_marijuana_problems | 19.20 |
| esi_marijuana_use | ~~ | esi_drug_use | 8.75 |
| esi_marijuana_use | ~~ | esi_drug_problems | 6.49 |
| esi_marijuana_use | ~~ | esi_alcohol_use | 4.21 |
| esi_marijuana_use | ~~ | esi_alcohol_problems | 7.16 |
| esi_marijuana_problems | ~~ | esi_drug_use | 4.37 |
| esi_marijuana_problems | ~~ | esi_drug_problems | 1.14 |
| esi_marijuana_problems | ~~ | esi_alcohol_use | 2.33 |
| esi_marijuana_problems | ~~ | esi_alcohol_problems | 0.49 |
| esi_drug_use | ~~ | esi_drug_problems | 0.02 |
| esi_drug_use | ~~ | esi_alcohol_use | 0.00 |
| esi_drug_use | ~~ | esi_alcohol_problems | 1.95 |
| esi_drug_problems | ~~ | esi_alcohol_use | 1.95 |
| esi_drug_problems | ~~ | esi_alcohol_problems | 4.27 |
| esi_alcohol_use | ~~ | esi_alcohol_problems | 17.97 |
