# Supplementary material for: Exploring the psychometric properties of the externalizing spectrum inventory-brief form in a Swedish forensic psychiatric inpatient sample
Source: BMC Psychiatry. 2023 Mar 21;23:184. doi: 10.1186/s12888-023-04609-y (PMC10031895; doi:10.1186/s12888-023-04609-y)
Supplement: Supplementary file 8 — Supplementary Material 8 Modification indices of the bifactor model [file 12888_2023_4609_MOESM8_ESM.docx]

**Supplementary Material 8. Modification indices of the bifactor model.**

| Left hand | Operator | Right hand | Modification index |
| --- | --- | --- | --- |
| callous_aggression | =~ | esi_problematic_impulsivity | 1.87 |
| callous_aggression | =~ | esi_theft | 2.37 |
| callous_aggression | =~ | esi_irresponsibility | 0.27 |
| callous_aggression | =~ | esi_marijuana_use | 2.60 |
| callous_aggression | =~ | esi_marijuana_problems | 0.23 |
| callous_aggression | =~ | esi_drug_use | 0.11 |
| callous_aggression | =~ | esi_drug_problems | 2.84 |
| callous_aggression | =~ | esi_alcohol_use | 1.20 |
| callous_aggression | =~ | esi_alcohol_problems | 1.33 |
| substance_abuse | =~ | esi_impatient_urgency | 4.14 |
| substance_abuse | =~ | esi_dependability | 0.27 |
| substance_abuse | =~ | esi_fraud | 0.09 |
| substance_abuse | =~ | esi_alienation | 1.13 |
| substance_abuse | =~ | esi_boredom_proneness | 0.81 |
| substance_abuse | =~ | esi_planful_control | 2.80 |
| substance_abuse | =~ | esi_blame_externalization | 0.00 |
| substance_abuse | =~ | esi_honesty | 0.03 |
| substance_abuse | =~ | esi_rebelliousness | 9.27 |
| substance_abuse | =~ | esi_physical_aggression | 2.13 |
| substance_abuse | =~ | esi_destructive_aggression | 1.35 |
| substance_abuse | =~ | esi_relational_aggression | 3.95 |
| substance_abuse | =~ | esi_empathy | 0.06 |
| substance_abuse | =~ | esi_excitement_seeking | 0.87 |
| callous_aggression | ~~ | substance_abuse | 0.32 |
| esi_problematic_impulsivity | ~~ | esi_theft | 0.56 |
| esi_problematic_impulsivity | ~~ | esi_irresponsibility | 0.08 |
| esi_problematic_impulsivity | ~~ | esi_impatient_urgency | 4.10 |
| esi_problematic_impulsivity | ~~ | esi_dependability | 1.33 |
| esi_problematic_impulsivity | ~~ | esi_fraud | 3.04 |
| esi_problematic_impulsivity | ~~ | esi_alienation | 1.06 |
| esi_problematic_impulsivity | ~~ | esi_boredom_proneness | 0.28 |
| esi_problematic_impulsivity | ~~ | esi_planful_control | 22.41 |
| esi_problematic_impulsivity | ~~ | esi_blame_externalization | 0.00 |
| esi_problematic_impulsivity | ~~ | esi_honesty | 0.04 |
| esi_problematic_impulsivity | ~~ | esi_rebelliousness | 0.12 |
| esi_problematic_impulsivity | ~~ | esi_physical_aggression | 0.20 |
| esi_problematic_impulsivity | ~~ | esi_destructive_aggression | 1.48 |
| esi_problematic_impulsivity | ~~ | esi_relational_aggression | 0.17 |
| esi_problematic_impulsivity | ~~ | esi_empathy | 1.58 |
| esi_problematic_impulsivity | ~~ | esi_excitement_seeking | 0.01 |
| esi_problematic_impulsivity | ~~ | esi_marijuana_use | 4.74 |
| esi_problematic_impulsivity | ~~ | esi_marijuana_problems | 2.08 |
| esi_problematic_impulsivity | ~~ | esi_drug_use | 8.61 |
| esi_problematic_impulsivity | ~~ | esi_drug_problems | 0.12 |
| esi_problematic_impulsivity | ~~ | esi_alcohol_use | 3.93 |
| esi_problematic_impulsivity | ~~ | esi_alcohol_problems | 0.40 |
| esi_theft | ~~ | esi_irresponsibility | 0.09 |
| esi_theft | ~~ | esi_impatient_urgency | 3.81 |
| esi_theft | ~~ | esi_dependability | 0.85 |
| esi_theft | ~~ | esi_fraud | 6.06 |
| esi_theft | ~~ | esi_alienation | 3.71 |
| esi_theft | ~~ | esi_boredom_proneness | 1.22 |
| esi_theft | ~~ | esi_planful_control | 0.64 |
| esi_theft | ~~ | esi_blame_externalization | 0.09 |
| esi_theft | ~~ | esi_honesty | 0.46 |
| esi_theft | ~~ | esi_rebelliousness | 1.37 |
| esi_theft | ~~ | esi_physical_aggression | 0.14 |
| esi_theft | ~~ | esi_destructive_aggression | 6.61 |
| esi_theft | ~~ | esi_relational_aggression | 0.18 |
| esi_theft | ~~ | esi_empathy | 2.67 |
| esi_theft | ~~ | esi_excitement_seeking | 0.03 |
| esi_theft | ~~ | esi_marijuana_use | 0.00 |
| esi_theft | ~~ | esi_marijuana_problems | 0.04 |
| esi_theft | ~~ | esi_drug_use | 0.50 |
| esi_theft | ~~ | esi_drug_problems | 0.70 |
| esi_theft | ~~ | esi_alcohol_use | 0.07 |
| esi_theft | ~~ | esi_alcohol_problems | 0.39 |
| esi_irresponsibility | ~~ | esi_impatient_urgency | 0.05 |
| esi_irresponsibility | ~~ | esi_dependability | 3.25 |
| esi_irresponsibility | ~~ | esi_fraud | 7.81 |
| esi_irresponsibility | ~~ | esi_alienation | 1.03 |
| esi_irresponsibility | ~~ | esi_boredom_proneness | 2.07 |
| esi_irresponsibility | ~~ | esi_planful_control | 0.64 |
| esi_irresponsibility | ~~ | esi_blame_externalization | 0.40 |
| esi_irresponsibility | ~~ | esi_honesty | 0.33 |
| esi_irresponsibility | ~~ | esi_rebelliousness | 0.06 |
| esi_irresponsibility | ~~ | esi_physical_aggression | 5.82 |
| esi_irresponsibility | ~~ | esi_destructive_aggression | 0.07 |
| esi_irresponsibility | ~~ | esi_relational_aggression | 0.07 |
| esi_irresponsibility | ~~ | esi_empathy | 1.29 |
| esi_irresponsibility | ~~ | esi_excitement_seeking | 0.49 |
| esi_irresponsibility | ~~ | esi_marijuana_use | 0.35 |
| esi_irresponsibility | ~~ | esi_marijuana_problems | 0.29 |
| esi_irresponsibility | ~~ | esi_drug_use | 0.02 |
| esi_irresponsibility | ~~ | esi_drug_problems | 0.59 |
| esi_irresponsibility | ~~ | esi_alcohol_use | 1.03 |
| esi_irresponsibility | ~~ | esi_alcohol_problems | 0.83 |
| esi_impatient_urgency | ~~ | esi_dependability | 0.03 |
| esi_impatient_urgency | ~~ | esi_fraud | 3.50 |
| esi_impatient_urgency | ~~ | esi_alienation | 0.54 |
| esi_impatient_urgency | ~~ | esi_boredom_proneness | 4.42 |
| esi_impatient_urgency | ~~ | esi_planful_control | 2.00 |
| esi_impatient_urgency | ~~ | esi_blame_externalization | 0.63 |
| esi_impatient_urgency | ~~ | esi_honesty | 0.16 |
| esi_impatient_urgency | ~~ | esi_rebelliousness | 1.05 |
| esi_impatient_urgency | ~~ | esi_physical_aggression | 0.00 |
| esi_impatient_urgency | ~~ | esi_destructive_aggression | 0.31 |
| esi_impatient_urgency | ~~ | esi_relational_aggression | 0.28 |
| esi_impatient_urgency | ~~ | esi_empathy | 0.70 |
| esi_impatient_urgency | ~~ | esi_excitement_seeking | 2.65 |
| esi_impatient_urgency | ~~ | esi_marijuana_use | 0.06 |
| esi_impatient_urgency | ~~ | esi_marijuana_problems | 1.91 |
| esi_impatient_urgency | ~~ | esi_drug_use | 0.74 |
| esi_impatient_urgency | ~~ | esi_drug_problems | 0.06 |
| esi_impatient_urgency | ~~ | esi_alcohol_use | 1.07 |
| esi_impatient_urgency | ~~ | esi_alcohol_problems | 0.06 |
| esi_dependability | ~~ | esi_fraud | 1.60 |
| esi_dependability | ~~ | esi_alienation | 0.32 |
| esi_dependability | ~~ | esi_boredom_proneness | 0.47 |
| esi_dependability | ~~ | esi_planful_control | 4.69 |
| esi_dependability | ~~ | esi_blame_externalization | 1.35 |
| esi_dependability | ~~ | esi_honesty | 8.49 |
| esi_dependability | ~~ | esi_rebelliousness | 1.58 |
| esi_dependability | ~~ | esi_physical_aggression | 0.85 |
| esi_dependability | ~~ | esi_destructive_aggression | 0.61 |
| esi_dependability | ~~ | esi_relational_aggression | 3.42 |
| esi_dependability | ~~ | esi_empathy | 1.15 |
| esi_dependability | ~~ | esi_excitement_seeking | 0.29 |
| esi_dependability | ~~ | esi_marijuana_use | 0.01 |
| esi_dependability | ~~ | esi_marijuana_problems | 6.77 |
| esi_dependability | ~~ | esi_drug_use | 3.21 |
| esi_dependability | ~~ | esi_drug_problems | 0.60 |
| esi_dependability | ~~ | esi_alcohol_use | 0.71 |
| esi_dependability | ~~ | esi_alcohol_problems | 6.77 |
| esi_fraud | ~~ | esi_alienation | 0.22 |
| esi_fraud | ~~ | esi_boredom_proneness | 2.22 |
| esi_fraud | ~~ | esi_planful_control | 5.22 |
| esi_fraud | ~~ | esi_blame_externalization | 0.14 |
| esi_fraud | ~~ | esi_honesty | 0.29 |
| esi_fraud | ~~ | esi_rebelliousness | 0.52 |
| esi_fraud | ~~ | esi_physical_aggression | 2.97 |
| esi_fraud | ~~ | esi_destructive_aggression | 4.85 |
| esi_fraud | ~~ | esi_relational_aggression | 4.27 |
| esi_fraud | ~~ | esi_empathy | 1.33 |
| esi_fraud | ~~ | esi_excitement_seeking | 0.62 |
| esi_fraud | ~~ | esi_marijuana_use | 1.37 |
| esi_fraud | ~~ | esi_marijuana_problems | 0.13 |
| esi_fraud | ~~ | esi_drug_use | 0.10 |
| esi_fraud | ~~ | esi_drug_problems | 0.49 |
| esi_fraud | ~~ | esi_alcohol_use | 0.36 |
| esi_fraud | ~~ | esi_alcohol_problems | 0.09 |
| esi_alienation | ~~ | esi_boredom_proneness | 0.82 |
| esi_alienation | ~~ | esi_planful_control | 0.50 |
| esi_alienation | ~~ | esi_blame_externalization | 2.41 |
| esi_alienation | ~~ | esi_honesty | 0.08 |
| esi_alienation | ~~ | esi_rebelliousness | 0.04 |
| esi_alienation | ~~ | esi_physical_aggression | 0.21 |
| esi_alienation | ~~ | esi_destructive_aggression | 1.18 |
| esi_alienation | ~~ | esi_relational_aggression | 2.38 |
| esi_alienation | ~~ | esi_empathy | 2.46 |
| esi_alienation | ~~ | esi_excitement_seeking | 2.27 |
| esi_alienation | ~~ | esi_marijuana_use | 0.05 |
| esi_alienation | ~~ | esi_marijuana_problems | 0.04 |
| esi_alienation | ~~ | esi_drug_use | 0.26 |
| esi_alienation | ~~ | esi_drug_problems | 1.05 |
| esi_alienation | ~~ | esi_alcohol_use | 0.01 |
| esi_alienation | ~~ | esi_alcohol_problems | 0.20 |
| esi_boredom_proneness | ~~ | esi_planful_control | 0.29 |
| esi_boredom_proneness | ~~ | esi_blame_externalization | 0.83 |
| esi_boredom_proneness | ~~ | esi_honesty | 3.57 |
| esi_boredom_proneness | ~~ | esi_rebelliousness | 0.04 |
| esi_boredom_proneness | ~~ | esi_physical_aggression | 0.00 |
| esi_boredom_proneness | ~~ | esi_destructive_aggression | 0.16 |
| esi_boredom_proneness | ~~ | esi_relational_aggression | 2.91 |
| esi_boredom_proneness | ~~ | esi_empathy | 1.82 |
| esi_boredom_proneness | ~~ | esi_excitement_seeking | 6.42 |
| esi_boredom_proneness | ~~ | esi_marijuana_use | 0.26 |
| esi_boredom_proneness | ~~ | esi_marijuana_problems | 2.44 |
| esi_boredom_proneness | ~~ | esi_drug_use | 0.03 |
| esi_boredom_proneness | ~~ | esi_drug_problems | 0.79 |
| esi_boredom_proneness | ~~ | esi_alcohol_use | 0.76 |
| esi_boredom_proneness | ~~ | esi_alcohol_problems | 0.01 |
| esi_planful_control | ~~ | esi_blame_externalization | 1.46 |
| esi_planful_control | ~~ | esi_honesty | 4.48 |
| esi_planful_control | ~~ | esi_rebelliousness | 1.99 |
| esi_planful_control | ~~ | esi_physical_aggression | 1.22 |
| esi_planful_control | ~~ | esi_destructive_aggression | 0.04 |
| esi_planful_control | ~~ | esi_relational_aggression | 4.62 |
| esi_planful_control | ~~ | esi_empathy | 0.04 |
| esi_planful_control | ~~ | esi_excitement_seeking | 1.61 |
| esi_planful_control | ~~ | esi_marijuana_use | 2.37 |
| esi_planful_control | ~~ | esi_marijuana_problems | 0.11 |
| esi_planful_control | ~~ | esi_drug_use | 1.36 |
| esi_planful_control | ~~ | esi_drug_problems | 1.49 |
| esi_planful_control | ~~ | esi_alcohol_use | 0.58 |
| esi_planful_control | ~~ | esi_alcohol_problems | 0.02 |
| esi_blame_externalization | ~~ | esi_honesty | 0.84 |
| esi_blame_externalization | ~~ | esi_rebelliousness | 0.17 |
| esi_blame_externalization | ~~ | esi_physical_aggression | 2.13 |
| esi_blame_externalization | ~~ | esi_destructive_aggression | 1.18 |
| esi_blame_externalization | ~~ | esi_relational_aggression | 1.35 |
| esi_blame_externalization | ~~ | esi_empathy | 1.49 |
| esi_blame_externalization | ~~ | esi_excitement_seeking | 0.21 |
| esi_blame_externalization | ~~ | esi_marijuana_use | 0.81 |
| esi_blame_externalization | ~~ | esi_marijuana_problems | 0.06 |
| esi_blame_externalization | ~~ | esi_drug_use | 0.27 |
| esi_blame_externalization | ~~ | esi_drug_problems | 0.83 |
| esi_blame_externalization | ~~ | esi_alcohol_use | 0.01 |
| esi_blame_externalization | ~~ | esi_alcohol_problems | 0.00 |
| esi_honesty | ~~ | esi_rebelliousness | 0.39 |
| esi_honesty | ~~ | esi_physical_aggression | 2.36 |
| esi_honesty | ~~ | esi_destructive_aggression | 2.46 |
| esi_honesty | ~~ | esi_relational_aggression | 0.14 |
| esi_honesty | ~~ | esi_empathy | 0.02 |
| esi_honesty | ~~ | esi_excitement_seeking | 0.02 |
| esi_honesty | ~~ | esi_marijuana_use | 2.16 |
| esi_honesty | ~~ | esi_marijuana_problems | 1.41 |
| esi_honesty | ~~ | esi_drug_use | 2.39 |
| esi_honesty | ~~ | esi_drug_problems | 0.09 |
| esi_honesty | ~~ | esi_alcohol_use | 4.26 |
| esi_honesty | ~~ | esi_alcohol_problems | 0.69 |
| esi_rebelliousness | ~~ | esi_physical_aggression | 1.11 |
| esi_rebelliousness | ~~ | esi_destructive_aggression | 0.29 |
| esi_rebelliousness | ~~ | esi_relational_aggression | 1.31 |
| esi_rebelliousness | ~~ | esi_empathy | 0.47 |
| esi_rebelliousness | ~~ | esi_excitement_seeking | 3.07 |
| esi_rebelliousness | ~~ | esi_marijuana_use | 1.09 |
| esi_rebelliousness | ~~ | esi_marijuana_problems | 0.10 |
| esi_rebelliousness | ~~ | esi_drug_use | 2.83 |
| esi_rebelliousness | ~~ | esi_drug_problems | 3.69 |
| esi_rebelliousness | ~~ | esi_alcohol_use | 0.82 |
| esi_rebelliousness | ~~ | esi_alcohol_problems | 0.35 |
| esi_physical_aggression | ~~ | esi_destructive_aggression | 0.01 |
| esi_physical_aggression | ~~ | esi_relational_aggression | 0.81 |
| esi_physical_aggression | ~~ | esi_empathy | 4.48 |
| esi_physical_aggression | ~~ | esi_excitement_seeking | 0.16 |
| esi_physical_aggression | ~~ | esi_marijuana_use | 0.00 |
| esi_physical_aggression | ~~ | esi_marijuana_problems | 0.02 |
| esi_physical_aggression | ~~ | esi_drug_use | 0.04 |
| esi_physical_aggression | ~~ | esi_drug_problems | 3.79 |
| esi_physical_aggression | ~~ | esi_alcohol_use | 1.02 |
| esi_physical_aggression | ~~ | esi_alcohol_problems | 0.36 |
| esi_destructive_aggression | ~~ | esi_relational_aggression | 2.49 |
| esi_destructive_aggression | ~~ | esi_empathy | 0.01 |
| esi_destructive_aggression | ~~ | esi_excitement_seeking | 1.39 |
| esi_destructive_aggression | ~~ | esi_marijuana_use | 2.82 |
| esi_destructive_aggression | ~~ | esi_marijuana_problems | 0.31 |
| esi_destructive_aggression | ~~ | esi_drug_use | 0.20 |
| esi_destructive_aggression | ~~ | esi_drug_problems | 0.85 |
| esi_destructive_aggression | ~~ | esi_alcohol_use | 0.12 |
| esi_destructive_aggression | ~~ | esi_alcohol_problems | 1.11 |
| esi_relational_aggression | ~~ | esi_empathy | 3.36 |
| esi_relational_aggression | ~~ | esi_excitement_seeking | 5.60 |
| esi_relational_aggression | ~~ | esi_marijuana_use | 1.12 |
| esi_relational_aggression | ~~ | esi_marijuana_problems | 0.24 |
| esi_relational_aggression | ~~ | esi_drug_use | 0.05 |
| esi_relational_aggression | ~~ | esi_drug_problems | 0.05 |
| esi_relational_aggression | ~~ | esi_alcohol_use | 0.55 |
| esi_relational_aggression | ~~ | esi_alcohol_problems | 1.58 |
| esi_empathy | ~~ | esi_excitement_seeking | 0.32 |
| esi_empathy | ~~ | esi_marijuana_use | 2.67 |
| esi_empathy | ~~ | esi_marijuana_problems | 0.20 |
| esi_empathy | ~~ | esi_drug_use | 0.98 |
| esi_empathy | ~~ | esi_drug_problems | 2.19 |
| esi_empathy | ~~ | esi_alcohol_use | 0.01 |
| esi_empathy | ~~ | esi_alcohol_problems | 0.02 |
| esi_excitement_seeking | ~~ | esi_marijuana_use | 0.20 |
| esi_excitement_seeking | ~~ | esi_marijuana_problems | 0.48 |
| esi_excitement_seeking | ~~ | esi_drug_use | 3.36 |
| esi_excitement_seeking | ~~ | esi_drug_problems | 2.03 |
| esi_excitement_seeking | ~~ | esi_alcohol_use | 0.04 |
| esi_excitement_seeking | ~~ | esi_alcohol_problems | 3.29 |
| esi_marijuana_use | ~~ | esi_marijuana_problems | 11.44 |
| esi_marijuana_use | ~~ | esi_drug_use | 0.03 |
| esi_marijuana_use | ~~ | esi_drug_problems | 4.16 |
| esi_marijuana_use | ~~ | esi_alcohol_use | 1.98 |
| esi_marijuana_use | ~~ | esi_alcohol_problems | 1.11 |
| esi_marijuana_problems | ~~ | esi_drug_use | 6.79 |
| esi_marijuana_problems | ~~ | esi_drug_problems | 0.10 |
| esi_marijuana_problems | ~~ | esi_alcohol_use | 0.80 |
| esi_marijuana_problems | ~~ | esi_alcohol_problems | 0.09 |
| esi_drug_use | ~~ | esi_drug_problems | 3.79 |
| esi_drug_use | ~~ | esi_alcohol_use | 0.80 |
| esi_drug_use | ~~ | esi_alcohol_problems | 0.13 |
| esi_drug_problems | ~~ | esi_alcohol_use | 1.94 |
| esi_drug_problems | ~~ | esi_alcohol_problems | 1.71 |
| esi_alcohol_use | ~~ | esi_alcohol_problems | 16.48 |
